# Catalytic activity of nickel nanoparticles stabilized by adsorbing polymers for enhanced carbon sequestration

**DOI:** 10.1038/s41598-018-29605-1

**Published:** 2018-08-06

**Authors:** Seokju Seo, Gabriela Alvarez Perez, Ketan Tewari, Xavier Comas, Myeongsub Kim

**Affiliations:** 1 Department of Ocean and Mechanical Engineering, Florida Atlantic University, 777 Glades Road, Boca Raton, FL33431 USA; 2 Department of Mechanical Engineering, Nirma University, Sarkhej-Gandhinagar Highway, Chandlodia, Gota, Ahmedabad, Gujarat 382481 India; 3 Department of Geosciences, Florida Atlantic University, 777 Glades Road, Boca Raton, FL33431 USA

## Abstract

This work shows the potential of nickel (Ni) nanoparticles (NPs) stabilized by polymers for accelerating carbon dioxide (CO_2_) dissolution into saline aquifers. The catalytic characteristics of Ni NPs were investigated by monitoring changes in diameter of CO_2_ microbubbles. An increase in ionic strength considerably reduces an electrostatic repulsive force in pristine Ni NPs, thereby decreasing their catalytic potential. This study shows how cationic dextran (DEX), nonionic poly(vinyl pyrrolidone) (PVP), and anionic carboxy methylcellulose (CMC) polymers, the dispersive behaviors of Ni NPs can be used to overcome the negative impact of salinity on CO_2_ dissolution. The cationic polymer, DEX was less adsorbed onto NPs surfaces, thereby limiting the Ni NPs’ catalytic activity. This behavior is due to a competition for Ni NPs’ surface sites between the cation and DEX under high salinity. On the other hand, the non/anionic polymers, PVP and CMC could be relatively easily adsorbed onto anchoring sites of Ni NPs by the monovalent cation, Na^+^. Considerable dispersion of Ni NPs by an optimal concentration of the anionic polymers improved their catalytic capabilities even under unfavorable conditions for CO_2_ dissolution. This study has implications for enhancing geologic sequestration into deep saline aquifers for the purposes of mitigating atmospheric CO_2_ levels.

## Introduction

Greenhouse gas emissions attributed to anthropogenic activities have increased significantly over the past century and continue to escalate. This increase causes measurable impacts on global warming, leading to climate-related concerns such as the unprecedented rising of sea levels along with more frequent and intense wildfires, floods, droughts, and tropical storms. Such adverse effects not only disrupt natural ecosystems, but they also pose serious risks to human populations^1^. Carbon dioxide (CO_2_) from fossil fuel combustion and industrial processes has accounted for 78% of the total greenhouse gas emissions^2^. Although natural systems can absorb CO_2_ back as part of the carbon cycle, these natural uptake processes are not enough to offset the escalating amount of anthropogenic CO_2_ released into the atmosphere^3^. Mitigation of CO_2_ emissions is an imminent global concern and must be urgently addressed by both political and technological implementations. As a result, many strategies have been proposed to mitigate global atmospheric CO_2_ levels^4–6^.

One promising strategy is to inject CO_2_ into subsurface rock formations because they provide the largest storage potential^7–10^. Among various geological storage options (e.g., saline aquifers, depleted oil or natural gas reservoirs, and coalbed reservoirs), deep saline aquifers provide the highest potential capacity, with approximately 10,000 billion metric tons of carbon in total^11^. However, safe storage through a solubility trapping process is controversial since most of the injected CO_2_ remains as CO_2_ molecules in the absence of a catalyst for enhancing the reaction rate of carbonic acid (H_2_CO_3_) formation^12^. This reaction rate is further hindered due to a high salinity content in brine, as preferable saline concentrations for CO_2_ storage range under 5% weight per volume while some aquifers have salinities ranging up to 25%^13^. As a result, the strategy of the accelerated CO_2_ dissolution process should be investigated in order to achieve a more feasible means of storing CO_2_ into deep saline aquifers.

Engineered nanoparticles (NPs) have attracted much attention in numerous applications due to their unique material and surface properties^14–16^. In addition, a high surface to volume ratio of NPs can considerably enhance their interfacial reactivity with the surrounding medium^17^. In particular, nickel (Ni) NPs has the potential to accelerate the rate of CO_2_ dissolution, especially under unfavorable water chemistry^18–21^. Major catalysts for the hydration of CO_2_ include the enzyme carbonic anhydrase^22,23^, hypobromous acid (HOBr), hypochlorous acid (HClO)^24^ and boric acid (B(OH)_3_)^25,26^. However, these catalysts are effective primarily in the alkaline range. Especially, enzyme carbonic anhydrase at a pH less than 7 catalyzes the dehydration of the bicarbonate ion (HCO_3_^−^)^27^. Unlike these catalysts, the Ni NPs’ catalytic activity is pH independent^18,21^. Since many saline aquifers are in an acidic condition (e.g., pH 2.3–2.24 in Indiana County^28^, Pennsylvania, pH 4.2 in Indiana County, Pennsylvania^29^), the feasibility of metallic Ni NPs as a catalytic additive for the hydration of CO_2_ was tested in a wide range of pH to enhance carbon sequestration in deep saline aquifers^21^. Furthermore, Ni NPs can be an effective catalyst in deep saline aquifers due to superior corrosion resistance and resistance to high-temperature oxidation of Ni^30–32^ under reservoir-specific temperature conditions (e.g., 40 °C^10^ and 62 °C^33^). Although high ionic strengths by salinity contents in brine lead to a substantial decrease in Ni NPs’ catalytic activity^21^, we observed the possibility of Ni NPs to accelerate CO_2_ dissolution even in acidic brine at a moderate salinity. However, many reservoirs are characterized by extreme salinities up to 25% and the upper limit of the Ni NPs’ catalytic activity at various salinities is still uncertain. To maximize the CO_2_ storage capacity in geologic formations at high salinity and to test the feasibility of Ni NPs-assisted CO_2_ dissolution in the field scale, quantification of the catalytic behaviors of Ni NPs in wide-ranging ionic strengths should be accomplished.

In addition, because NPs are easily aggregated due to the strong potential of van der Waals force as salinity increases, finding an efficient way to disperse particles in a solution for their maximum performance is another critical step for fully utilizing the Ni NP’s potential in brine with an extreme salinity content. One of the most promising solutions to improve the Ni NPs’ catalytic activity is to stabilize them in polymer matrices. Without altering material properties of NPs, small molecule ligands in polymers are used to control stabilization of NPs against agglomeration^34,35^. While polymer matrices retain the inherent properties of pristine NPs, NPs-polymer interactions lead to an increase in potential of NPs as catalysts and processability of the polymer matrix^36^. As many publications have shown, NPs bonded by polymer ligands promote NPs’ catalytic reactions at their surface^37–39^. The most widely used host polymer materials include poly(vinyl pyrrolidone) (PVP)^40^ and carboxy methylcellulose (CMC)^41,42^, and the best performance of NPs’ catalytic behaviors in these polymers are generally determined based on the mechanical and thermal properties of the polymeric supports. Due to large variations in polymer properties that control the NPs’ catalytic activity, substantial uncertainties about the optimal combination of NPs and polymers remain. These uncertainties can be mitigated by a fast and simple approach, as shown in this study, that determines CO_2_ dissolution phenomena accurately.

An experimental bubble-based microfluidic approach has been used as one of the most promising and accurate methods to study the CO_2_ dissolution dynamics into aqueous solutions. CO_2_ dissolution into an aqueous phase occurring at the interface has been quantified successfully by observing bubble size and morphology^43–48^. Owing to the high surface area to volume ratio of microbubble, the mass transfer rate is considerably enhanced over very short time scales, resulting in fast changes in bubble size^48–54^. The rapid change in CO_2_ microbubbles facilitates accurate quantification of the CO_2_ dissolution rate. The time-dependent size shrinkage or expansion of CO_2_ bubbles is due mainly to a reversible reaction of absorption and desorption of CO_2_ associated with the properties of the surrounding liquid. When gaseous CO_2_ absorption into the aqueous phase dominates, the removal of CO_2_(g) trapped as a bubble leads to shrinkage of CO_2_ bubbles. On the other hand, as increasing desorption of CO_2_(aq) from the aqueous phase to the gaseous phase, bubble expansion occurs. In a similar way, the role of catalytic activity controlled by Ni NPs-polymer on CO_2_ dissolution can be also revealed by observation of microbubble morphology via a microfluidic technology.

**Figure 1:**
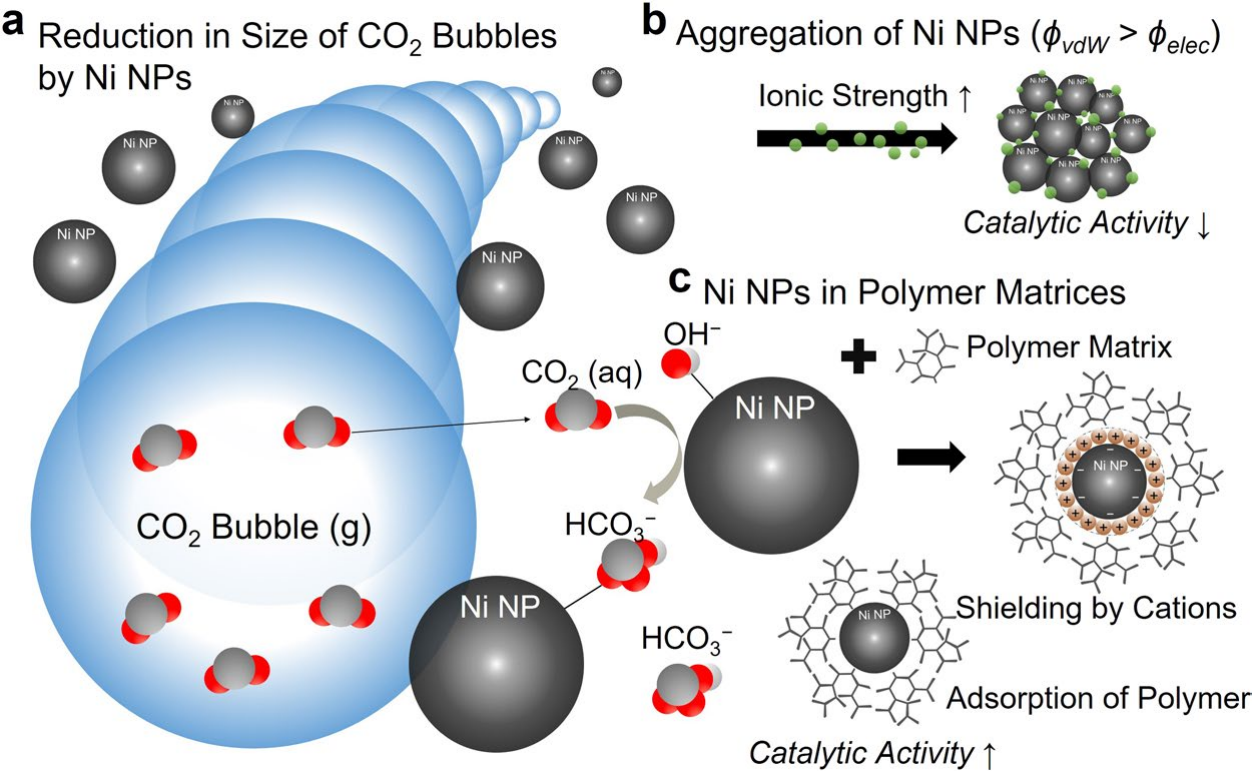
Conceptual illustration for the hydration of CO_2_. (**a**) Accelerated CO_2_ dissolution by Ni NPs catalytic potential. (**b**) The decreased catalytic potential of Ni NPs by the aggregation behavior of NPs in high ionic strengths. (**c**) Stabilized Ni NPs by polymers in brine at high salinity levels.

In the present work, we introduce a simple and time efficient microfluidic technique that tests the feasibility of utilizing Ni NPs in polymer matrices to accelerate gaseous CO_2_ dissolution into a continuous aqueous solution (Fig. 1). First, the time-dependent changes in CO_2_ microbubble size in response to the salinity concentrations with and without Ni NPs was quantified using high-speed imaging. The results offered an evidence of a decrease in catalytic potential as the salinity increases because of a Ni NPs’ aggregation behavior in brine. To further enhance Ni NPs’ catalytic potential, the hydration of CO_2_ was measured using Ni NPs stabilized with cationic dextran (DEX), nonionic PVP, and anionic CMC polymers. The results suggest that the Ni NPs-polymer matrix could enhance CO_2_ dissolution up to three times when compared to dissolution into a pure aqueous solution. Optimal concentrations of these polymers for enhancing Ni NPs performance were also determined to enhance carbon sequestration processes.

## Results

### Salinity effects on CO_2_ dissolution

**Figure 2:**
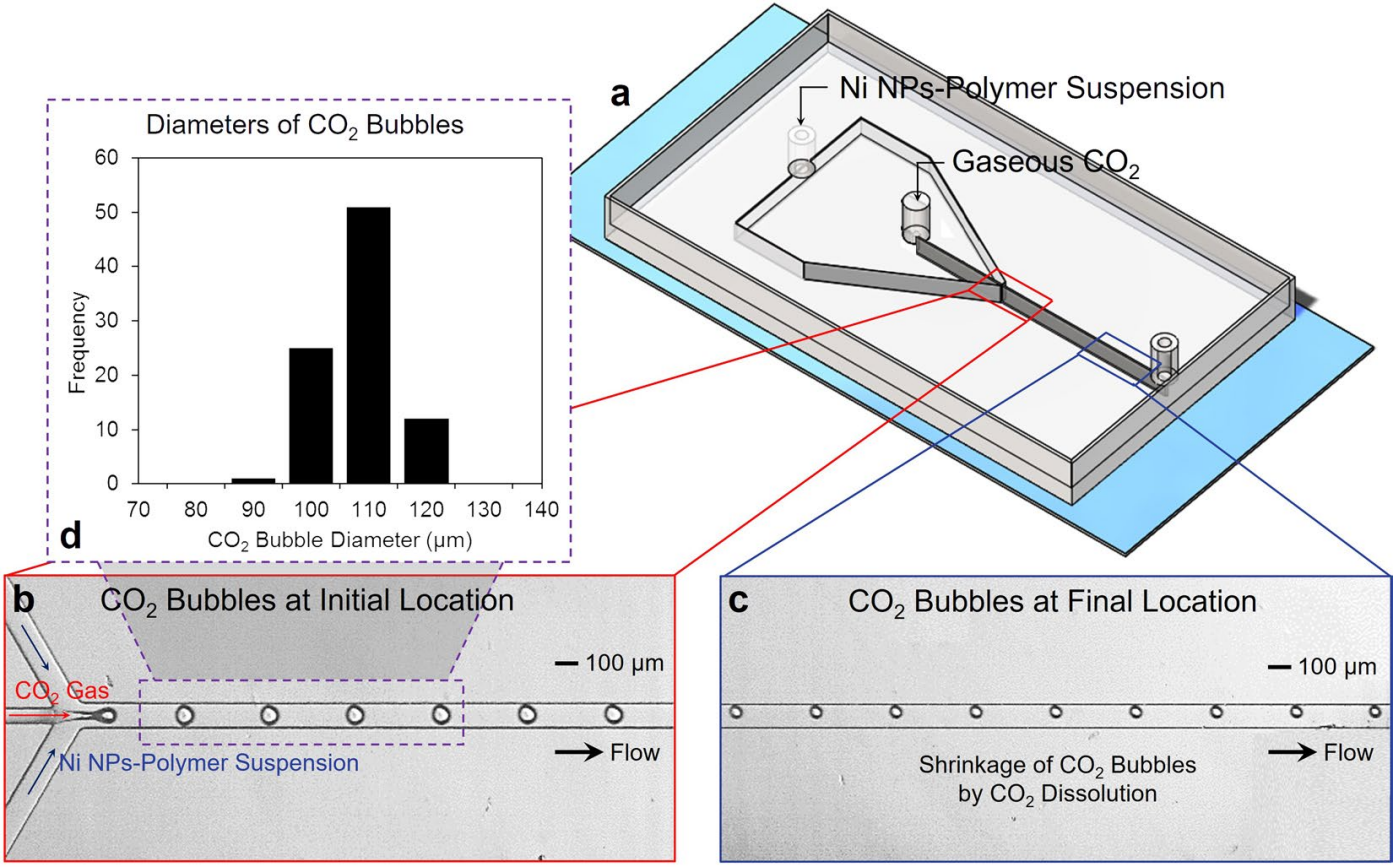
A microfluidic approach for evaluation of CO_2_ dissolution into water. (**a**) A microfluidic platform with its configuration. (**b**,**c**) Representative micrographs of CO_2_ bubbles at two different locations (near the junction and the outlet) in the flow-focusing geometry. (**d**) A histogram of estimated diameters of CO_2_ bubbles near the junction.

Figure 2a shows the schematic of a microfluidic device for investigation of CO_2_ dissolution into an aqueous solution. In a flow-focusing geometry, a gaseous CO_2_ was focused by the injected continuous aqueous phase on both sides. A combination of a shear force by the two aqueous streams and an interfacial tension force between the liquid and the gas phases generates spherical CO_2_ bubbles at the junction. Once the bubbles are generated, the mass transfer of gaseous CO_2_ immediately occurs at the gas-liquid interface while traveling downstream and subsequently changing their sizes. To enhance measurement accuracy, the initial bubble diameter and generation frequency of CO_2_ bubbles should be maintained constant (Fig. 2b). To achieve this goal, monodispersed bubbles were consistently generated in all experiments by fixing input flow rate and CO_2_ gas pressure at 0.3 mL min^−1^ and 1 psi, respectively. Figure 2d shows a representative distribution of the bubble size in one of our tests.

**Figure 3:**
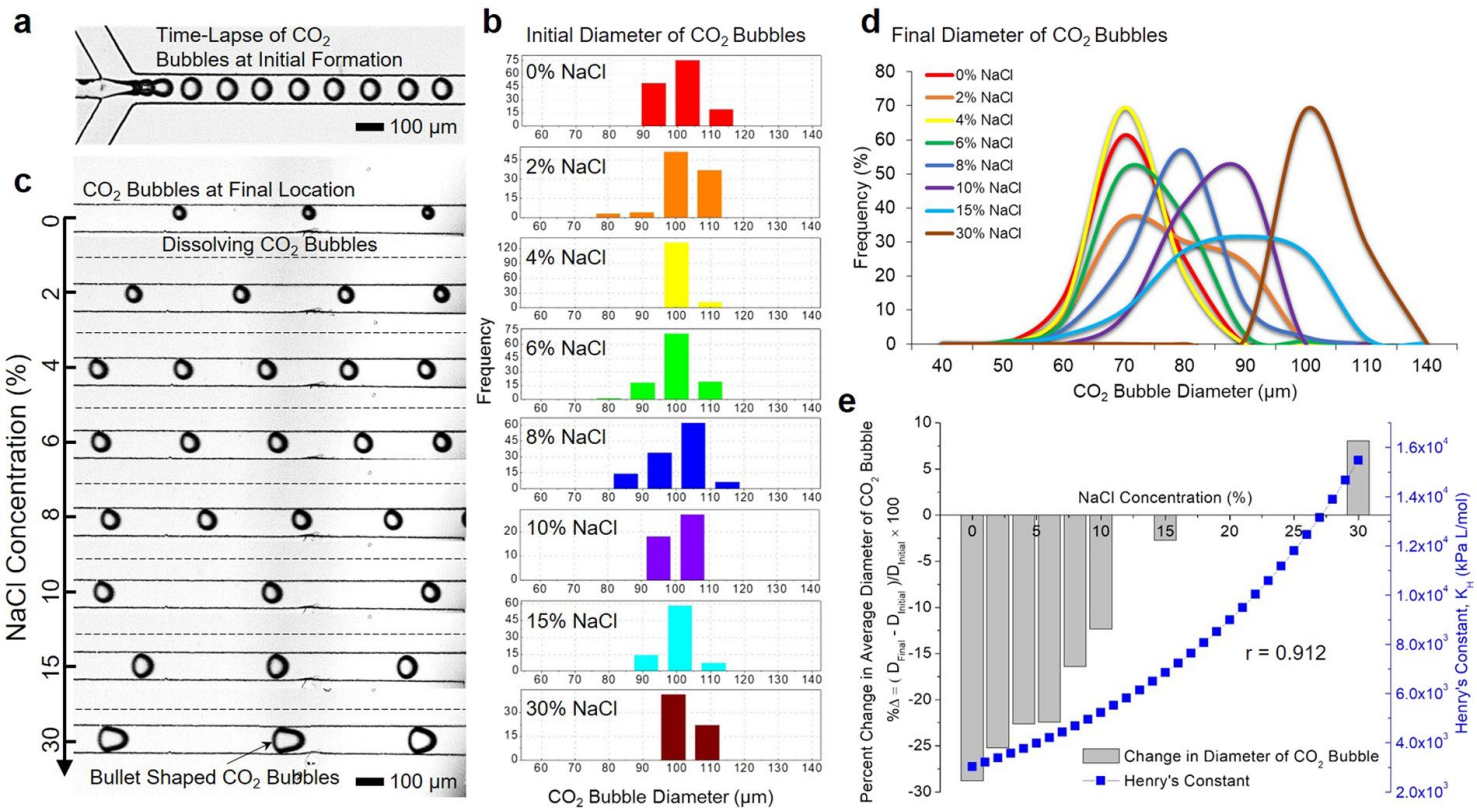
(**a**) A time series of CO_2_ bubbles at the junction of the microchannel. (**b**) Histograms of the initial diameter of CO_2_ bubbles. (**c**) Representative micrographs of CO_2_ bubbles near the outlet at the concentration of salinity (0%, 2%, 4%, 6%, 8%, 10%, 15%, and 30% NaCl). (**d**) Variations in size of CO_2_ bubbles near the outlet at different salinities. (**e**) A diagram of percent changes (%∆ = (*D*_*final*_ − *D*_*initial*_)/*D*_*initial*_ × 100) in average diameter of CO_2_ bubbles and the graph of Henry’s constant () at different salinities.

To evaluate the dissolution behaviors of CO_2_ at salinity, a series of tests was performed at 0~30% (weight per volume) NaCl solutions with neutral pH. It should be noted that pH is an important parameter to change the hydration of CO_2_ at different salinity levels^21,55^. Figure 3a shows a time series of CO_2_ bubbles near the junction in the microfluidic device. The velocity of CO_2_ bubbles in the microchannel was approximately 5.926 ± 0.92 mm s^−1^ and their residence time between two different locations (near junction and outlet) was 3.46 ± 0.54 s. Each CO_2_ bubble was generated at a constant interval of approximately 20 ms. A distribution of CO_2_ bubble diameters was determined for more than 50 CO_2_ bubbles collected for each experiment (Fig. 3b). Applying Gaussian, Lognormal or Lorentzian fittings in these histograms, the average diameters of CO_2_ bubbles near junction (the initial location) are determined to be 104.93, 103.96, 99.41, 101.8, 100.35, 99.08, 96.04, and 98.66 μm at 0%, 2%, 4%, 6%, 8%, 10%, 15%, and 30% salinity, respectively (Fig. S1). We observed no substantial changes in initial size at different salinities. However, due to an increase in ionic strength, the degree of shrinkage of CO_2_ bubbles decreases proportionally (Fig. 3c and Supplementary Movie S1).

The average sizes of CO_2_ bubbles near the outlet (the final location) decrease by 30.2, 26.21, 22.51, 22.81, 16.47, 12.22, and 2.63 μm from 0 to 15% NaCl, respectively (Fig. 3d). At 30% NaCl, the expansion of CO_2_ bubbles occurs, increasing by 7.94 μm. Bulletlike CO_2_ bubbles are observed since the width of expanded CO_2_ bubbles is greater than that of the microchannel (100 μm). Gas exchange at the interface between the CO_2_ bubble and the aqueous phase starts when the bubble is generated^52,56^. Gaseous CO_2_ molecules trapped in the bubble start to diffuse into the aqueous phase. Meanwhile, dissolved gas molecules including nitrogen and oxygen are simultaneously desorbed back to the CO_2_ bubble. While the absorption rate of CO_2_ gas molecules is decreased with an increase in salinity, the change in CO_2_ bubble diameter was decreased since the desorption rate of dissolved gas molecules remains constant. Because desorption of dissolved gases dominates at 30% NaCl, the expansion of CO_2_ bubbles was observed near the outlet (the final location). Collectively, the variation in size of CO_2_ bubbles at different salinities indicates that the extent of CO_2_ dissolution considerably decreases as increasing salinity concentration.

The effect of salinity on CO_2_ dissolution could be explained by Henry’s Law described as follows^57^

1 $${k}_{H}={{k}_{H}}^{0}\cdot {10}^{0.138I}=\frac{{P}_{C{O}_{2}}}{{[C{O}_{2}]}_{aq}},$$

where *k*_*H*_^0^ (*3*.*45* × *10*^*3*^ *kPa L mol*^−1^) is Henry’s constant for CO_2_ in water at 25 °C and *I* is the ionic strength ($I=0.5\sum _{i}{C}_{i}{{z}_{i}}^{2}$, *C*_*i*_ is the concentration of ion of type *i*, and *z*_*i*_ is its valence), [*CO*_2_]_*aq*_ is aqueous CO_2_ concentration, and *P*_*CO2*_ is the partial pressure of the gaseous CO_2_. As the ionic strength in the aqueous phase increases, the value of Henry’s constant increases as well, and thereby decreases CO_2_ dissolution. To evaluate the influence of the ionic strength on the change in CO_2_ bubble size, Henry’s constants at 0 to 30% salinity were determined (Fig. 3e ). Figure 3e also shows percent changes of CO_2_ bubbles diameter at the initial and final locations (%∆ = (*D*_*final*_ − *D*_*initial*_)/*D*_*initial*_ × 100). The Pearson’s correlation coefficient (*r* = 0.912) suggests that a significant correlation exists between the variation in size of CO_2_ bubbles and Henry’s constants. It should be noted that a critical value for correlation coefficient *r* at the α = 0.01 level is 0.834 (6 degrees of freedom).

### Ni NPs-assisted CO_2_ dissolution

Ni NPs can overcome the decreased dissolution of CO_2_ under high saline concentrations through their excellent catalytic potential^21^. When the aqueous phase containing Ni NPs surrounds CO_2_ bubbles that travel along the microchannel, the diameter of CO_2_ bubbles becomes decreased thanks to the accelerated hydration of gaseous CO_2_ by the Ni NPs’ catalytic activity. The relevant chemical reactions occur subsequently as follows:

2 $$Ni\,NP+{H}_{2}O\to Ni\,NP-O{H}^{-}+{H}^{+}$$

3 $$Ni\,NP-O{H}^{-}+C{O}_{2}(aq)\to Ni\,NP-HC{{O}_{3}}^{-}$$

4 $$Ni\,NP-HC{{O}_{3}}^{-}+{H}_{2}O\to Ni\,NP-{H}_{2}O+HC{{O}_{3}}^{-}$$

When Ni NPs are put into an aqueous solution, they react with H_2_O to generate hydroxyl (OH^−^) groups on NPs’ surfaces. Then, OH^−^ groups react with CO_2_(aq) to produce HCO_3_^−^ ions attached to the surface of Ni NPs. These HCO_3_^-^ ions are replaced by H_2_O. A series of these catalytic chemical reactions associated with Ni NPs promotes accelerated removal of CO_2_(aq) from the water that consequently catalyzes the hydration of CO_2_. It should be noted that when metallic Ni NPs are exposed to an oxidative environment, their catalytic performance for the hydration of CO_2_ can considerably decrease due to the formation of an oxide layer on Ni NPs’ surfaces^58^. However, according to previous studies^58,59^, oxidation of Ni NPs did not occur at an ambient temperature for two months^59^ and the initiation of oxidation occurred at approximately 300 °C^58^. Taking this fact into consideration, the temperature range of deep saline aquifers (e.g., 40 °C^10^ and 62 °C^33^) is unlikely to considerably inhibit the Ni NPs’ catalytic activity by oxidation.

**Figure 4:**
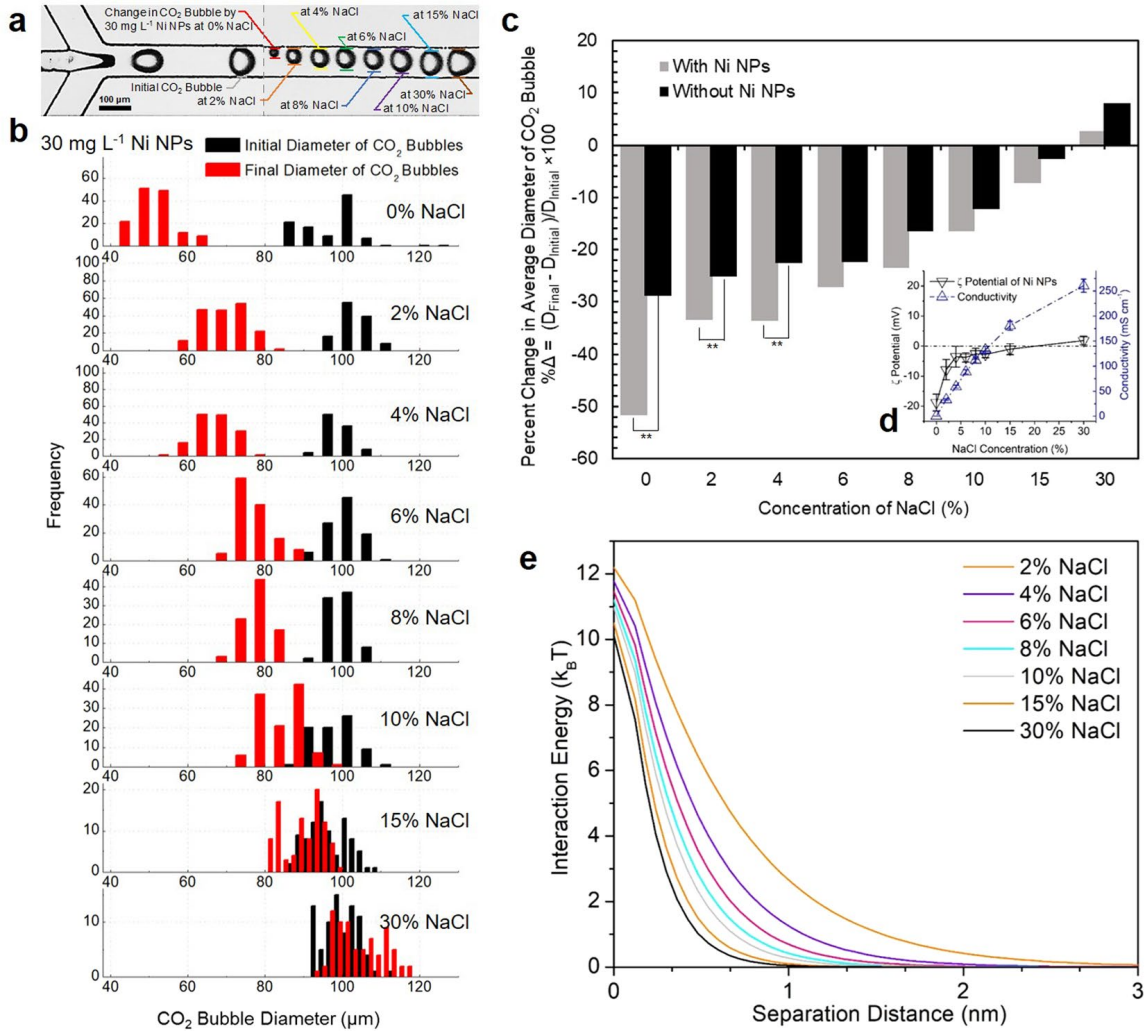
(**a**) A representative time-lapse microscopy image of CO_2_ bubbles near the junction and the outlet in the presence of Ni NPs at different salinities (0%, 2%, 4%, 6%, 8%, 10%, 15%, and 30% NaCl). (**b**) Histograms of sizes of CO_2_ bubbles near the junction (the initial location) and the outlet (the final location) to confirm the effect of salinity on Ni NPs’ catalytic potential. (**c**) The percent changes in average diameter of CO_2_ bubbles near the outlet in the presence and absence of Ni NPs at different salinities (**p <0.01). (**d**) The graphs of experimentally measured ζ potential and conductivity of Ni NPs solution at different salinities. (Error bars show mean ± standard error of the mean from three independent measurements). (**e**) The electrostatic double layer potential (*ϕ*_*elec*_) between two Ni NPs from the extended-DLVO theory at various ionic strengths.

Figure 4a shows representative time-lapse micrographs of CO_2_ bubbles in 30 mg L^−1^ concentration of Ni NPs solution under varying ionic strengths with neutral pH. The concentration of 30 mg L^−1^ Ni NPs that provides the maximum catalytic behavior of Ni NPs in pure water was derived from our previous research^21^. In the absence of salinity, a substantial decrease in diameter of CO_2_ bubbles was observed as a consequence of the catalytic potential of Ni NPs. As salinity increases, the change in CO_2_ bubble diameter was similar regardless of the presence of Ni NPs (Figs 4a and 3c). To understand the effect of salinity on Ni NPs’ catalytic potential, the changes on CO_2_ bubbles were determined at different salinities (Fig. 4b and Supplementary Movie S2). In the histogram, as salinity increases, the increase in diameter of CO_2_ bubbles near the outlet yields a shift to the right (). No considerable variation in initial size of CO_2_ bubbles (■, 101.64, 103.9, 98.96, 102.44, 100.55, 99.51, 95.87, 99.8 μm) was observed at 0%, 2%, 4%, 6%, 8%, 10%, 15%, and 30% salinity, respectively (Fig. S2). Figure 4c shows the percent changes (%∆ = (*D*_*final*_ − *D*_*initial*_)/*D*_*initial*_ × 100) in CO_2_ bubble diameter with −51.73 (−28.79), −33.45 (−25.21), −33.6 (−22.64), −27.13 (−22.42), −23.47 (−16.41), −16.41 (−12.33), −7.29 (−2.74), 2.71 (8.04)% in the presence (absence) of Ni NPs at the different ionic strengths, respectively. The difference in magnitude of these percent changes in CO_2_ bubble diameter represents Ni NPs’ catalytic potential for enhancement of the hydration of CO_2_. In the absence of salt, the percent change of CO_2_ bubbles in Ni NPs solution was approximately twofold higher than that without Ni NPs. However, an increase in NaCl concentrations results in a substantial decrease in Ni NPs’ catalytic potential due to their aggregation in high ionic suspension. Up to 4% NaCl, significant differences of the CO_2_ bubble size change with and without Ni NPs were confirmed by statistical estimations (*p* = 7.62 × 10^−18^, 6 × 10^−3^, 1.91 × 10^−5^ between the presence and absence of Ni NPs at 0~4% NaCl); however, no significant differences were estimated at greater than 6% NaCl regardless of Ni NPs (*p* = 6.5 × 10^−1^, 2.5 × 10^−1^, 6.57 × 10^−1^, 6.62 × 10^−2^, and 2.6 × 10^−2^ between the presence and absence of Ni NPs at 6~30% NaCl, respectively) (Fig. 4c).

The aggregation behavior of NPs at high salinity was verified by measurements of their zeta (ζ) potential at different salinities, as shown in Fig. 4d. According to Derjaguin, Landau, Verwey, and Overbeak (DLVO) theory^60,61^, an electrical double layer (EDL) of each colloidal particle generates a repulsion force to prevent particle-particle aggregation when particles approach each other. It is known that, if the ζ potential, electrical potential in EDL is less than ± 20 mV, particles are considered unstable and consequently aggregate and settle out of suspension. The average ζ potential of pristine Ni NPs in the absence of NaCl was measured to be −18.8 ± 2.8 mV. This suggests that Ni NPs in pure aqueous solution are still unstable. An increase in concentration of NaCl considerably changes the ζ potential to −3.75 ± 1.52 mV at the ionic strength of 1.02 M (6% NaCl). A plateau value of ζ potential was observed at ionic strengths of 1.37 M (8% NaCl) or greater, indicated as an electrolytic conductivity (Fig. 4d). This implies that the increase in ionic strength of suspension diminishes considerably the NPs stability, thereby decreasing their catalytic potential.

An extended DLVO model that accounts for two Ni NPs’ electrostatic double layer potential (*ϕ*_*elec*_) can support these results (Fig. 4e).

5 $${\varphi }_{{\rm{elec}}}=\,\frac{2\pi {e}_{0}\varepsilon r{\phi }^{2}\,\mathrm{ln}(1+{e}^{-h/{\kappa }^{-1}})}{{k}_{B}T},$$

where *e*_0_ is permittivity of free space (*8*.*85* × *10*^*−12*^ *F m*^*−1*^), *r* is a radius of Ni NPs (*50* *nm*), *ε* is a dimensionless dielectric constant of water (*80*.*1*), *φ* is the ζ potential of Ni NPs (*−18*.*8* *mV*), *e* is an electron charge (*1*.*6* × *10*^*−19*^ *coulomb*), *h* is the separation distance between two Ni NPs, *k*_*B*_ is the Boltzmann’s constant (*1*.*381* × *10*^*−23*^ *J K*^*−1*^), *T* is the absolute temperature (*298*.*15 K*). *κ*^*−1*^, which is the Debye length (or thickness of the double layer), and described as follows:

6 $${\kappa }^{-1}=\sqrt{\frac{{e}_{0}\varepsilon {k}_{B}T}{2{N}_{A}{e}^{2}I}\,}$$

where *e*_0_ is the permittivity of free space (*8*.*85 × 10*^*−12*^ *F m*^*−1*^), *ε* is the dimensionless dielectric constant of water (*80*.*1*) at 20 °C, *N*_*A*_ is the Avogadro’s number (*6*.*02 × 10*^23^* mol*^*−1*^), *e* is the electron charge (*1*.*6* × *10*^*−19*^ *coulomb*), and *I* is the ionic strength of solution (*mol m*^*−3*^). As the ionic strength in Ni NPs suspension increases, an energy barrier induced by two double layers of Ni NPs to prevent their aggregation considerably decreases since the Debye length is inversely proportional to the ionic strength of the solution. As a result, the electrostatic repulsive force becomes weaker and van der Waals attraction dominates, thereby becoming a favorable condition for particle-particle aggregation.

### Enhanced hydration of CO_2_ in Ni NPs-polymer solution

**Figure 5:**
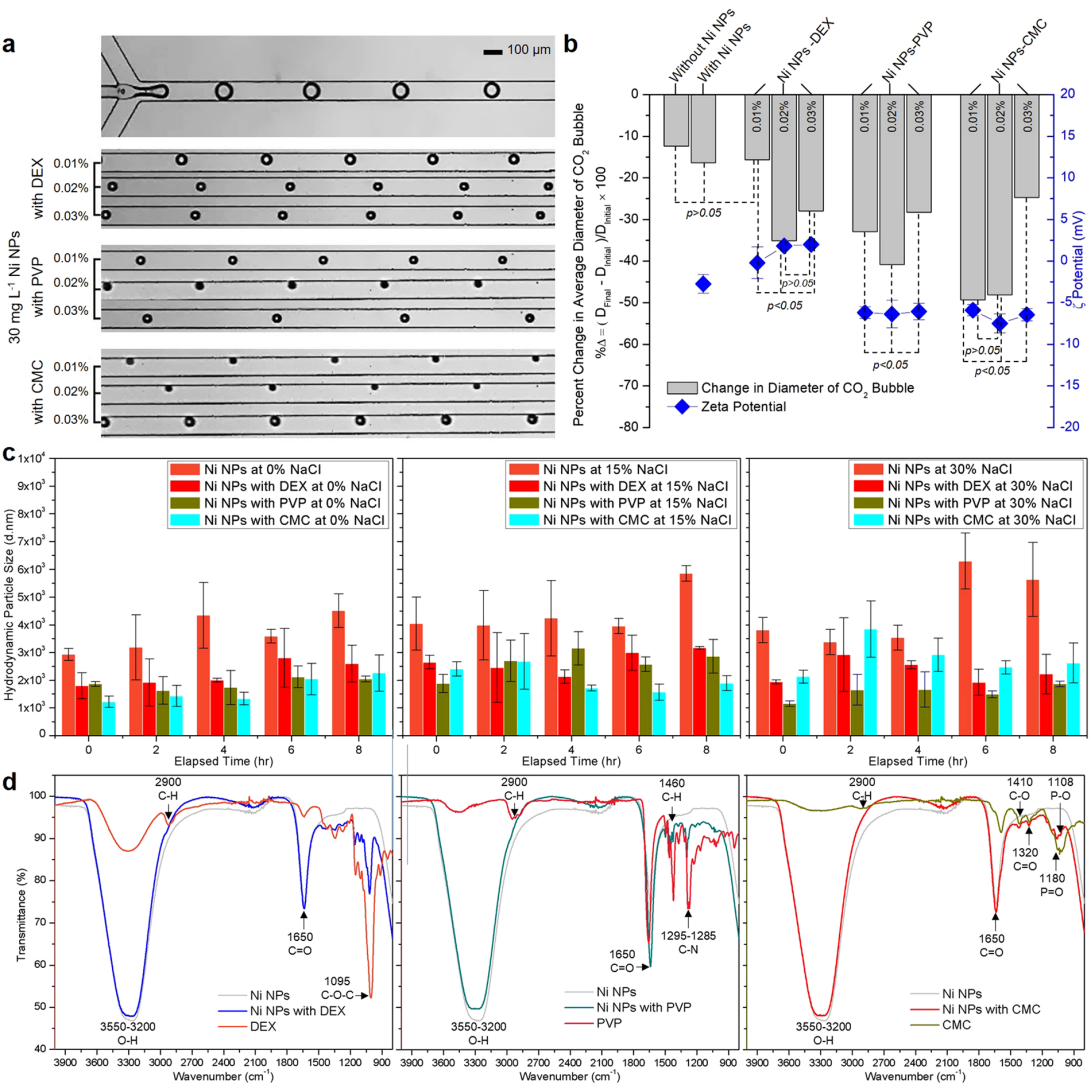
(**a**) Representative micrographs of CO_2_ bubbles near the junction (top) and the outlet (bottom) at stabilized Ni NPs by 0.01%, 0.02%, and 0.03% of DEX, PVP, and CMC at 10% NaCl. (**b**) A diagram of percent changes in average diameter of CO_2_ bubbles in Ni NPs solution with DEX, PVP, CMC and graphs () of measured ζ potential of stabilized Ni NPs as a function of polymer concentration at 0.01%, 0.02%, and 0.03%. (**c**) Diagrams of changes in hydrodynamic particle size (d.nm) of Ni NPs with 0.03% of DEX, PVP, and CMC at different salinities (0%, 15%, and 30% NaCl). (**d**) FTIR spectra of Ni NPs, polymers, and Ni NPs with DEX, PVP, and CMC.

Polymer matrices greatly stabilize NPs and therefore increase their catalytic potential^36^. The variations in diameter of CO_2_ bubbles were measured in 30 mg L^−1^ Ni NPs solution with the different types of polymers including DEX, PVP, and CMC, respectively. The salinity was chosen at 10% NaCl where the absolute value of ζ potential near zero (Fig. 4d) to find the optimal combination for the enhancement of steric stabilization of Ni NPs. Steric stabilization is a process in which NPs are prevented from the aggregation through the adsorption of macromolecules derived from polymers on the NPs surface. Figure 5a and Supplementary Movie S3 show representative images and videos of CO_2_ bubbles near the junction and the outlet. For each polymer, three different concentrations (0.01, 0.02, and 0.03%) were tested to evaluate the degree of bubble shrinkages.

Figure 5b shows the percent changes in CO_2_ bubble diameter under aforementioned conditions. For DEX, PVP, and CMC at 0.01%, 0.02%, and 0.03% concentrations, the initial diameters of CO_2_ bubbles were observed to be: 102.7, 106.37, 99.87, 103.9, 98.35, 105.57, 105.06, 97.5, and 104.6 μm, respectively (Fig. S3). The diameter changes for DEX, PVP, and CMC at 0.01%, 0.02%, and 0.03% were −15.65, −35.12, −27.99, −32.94, −40.85, −28.29, −49.31, −48.14, and −24.77%, respectively. With comparing the results at 10% NaCl without Ni NPs and polymer, Ni NPs at 10% NaCl (without polymers), Ni NPs with 0.01% DEX at 10% NaCl, no significant differences (*p* = 1.56 × 10^−1^ between Ni NPs and Ni NPs with 0.01% DEX at 10% NaCl) in the percent change in CO_2_ bubbles (−12.33, −16.41, and −15.65%, respectively) were observed. The cationic polymer molecules derived from DEX lead to increase in net surface charge of Ni NPs to be positive. However, 0.01% DEX was not considerably effective in increasing the ζ potential of Ni NPs in the presence of salinity. Stabilized Ni NPs by 0.01% DEX at 10% NaCl still possess almost zero net electrical charge on the surface (−0.2 mV, Fig. 5b). This observation can be explained as follows. The monovalent cation, Na^+^ and cationic polymeric molecules from DEX deposit on negatively charged Ni NPs’ surface due to the electrostatically mediated attraction. Under aqueous solutions enriched in the monovalent cation, there exists a competition for occupying Ni NPs’ surface sites between the monovalent cation and the cationic polymer. A large amount of Na^+^ ion in a solution is responsible for the decreased deposition of cationic polymer molecules on Ni NPs. As a result, cationic polymer molecules from DEX are less adsorbed at low concentration of DEX due to this competition. However, as the percentage of DEX contents in the polymer-Ni NPs mixture increases from 0.01% to 0.03%, more DEX polymer molecules adsorbed onto Ni NPs’ surface sites so that the ζ potential of Ni NPs was linearly increased from −0.2 to 2.0 mV (Fig. 5b). As a result, CO_2_ bubbles noticeably shrank to a smaller size at 0.02% and 0.03%.

Unlike cationic DEX polymer, the ζ potential of Ni NPs effectively decreased to −6.2 and −5.91 mV at even 0.01% concentration of PVP and CMC, respectively. This is because higher ionic strengths produce a shielding of negatively charged Ni NPs’ surface by a monovalent cation (Na^+^)^62,63^. Na^+^ ions attached on the NPs’ surface serve as anchoring sites so that the electrostatic repulsion force between Ni NPs and nonionic/anionic polymer molecules is decreased effectively^64^. Therefore, the stabilization of negatively charged Ni NPs in solution enriched by monovalent cations from NaCl, resulting in the accelerated hydration of CO_2_, attributes to the increase in ζ potential of Ni NPs. The increased catalytic potential of Ni NPs in PVP and CMC polymers reflects the large percent changes in CO_2_ bubbles diameter at −32.94 and −49.31%, respectively.

When the concentration of PVP was increased from 0.01 to 0.02%, the catalytic potential of Ni NPs was enhanced. As a result, the percent change in CO_2_ bubbles diameter was decreased from −32.94 to −40.85%, respectively. However, an increase in concentration of CMC from 0.01 to 0.02% is unlikely to be beneficial for steric stabilization of Ni NPs (Fig. 5b). There were insignificant differences (*p* = 9.43 × 10^−2^) in the effect of stabilized Ni NPs by 0.01 and 0.02% CMC on the percent change in CO_2_ bubbles size (−49.31 and −48.14%). As shown in the figure, while the decrease in ζ potential of Ni NPs in PVP and CMC was observed with no consequences, the Ni NPs’ catalytic activity was decreased considerably at 0.03% PVP and CMC. This is because the surplus polymer at 0.03% concentration has a tendency to create flocculation between NPs once the non/anionic polymer concentration is over the saturation of NPs dispersion. This result indicated that 0.02% PVP and 0.01% CMC are the optimal concentration for steric stabilization of Ni NPs.

To confirm the enhanced stability of Ni NPs by polymers, time-dependent changes in hydrodynamic Ni particle size were measured at different salinities (0%, 15%, 30% NaCl). Without polymers, initial average hydrodynamic sizes were 2929.33, 4040.5, 3810.5 nm at 0%, 15%, and 30% NaCl, respectively (Fig. 5c). However, Ni NPs with DEX, PVP and CMC showed a considerable decrease in size: 1800.8, 1866.33, 1216.2 nm at 0% NaCl, 2645, 1878, 2404.67 nm at 15% NaCl, and 1933.67, 1147.77, 2121.5 nm at 30% NaCl, respectively. Furthermore, while considerable changes in size were observed in pure Ni NPs after 8 hr (2929.33 → 4513, 4040.5 → 5855, 3810.5 → 5634 nm in 0%, 15%, and 30% NaCl, respectively), these changes were negligible in Ni NPs with polymers, suggesting that polymers could effectively increase the stability of Ni NPs.

General characterization of changes in Ni NPs’ properties associated with polymer adsorption and the specific interaction between Ni NPs and polymers could be further verified from the Fourier transform infrared spectroscopy (FTIR) spectra (Fig. 5d). Without polymers, a FTIR spectrum of Ni NPs has transmission bands at 3550-3200 cm^−1^ attributed to the O-H stretching region and 1650 cm^−1^ from the vibrations of C=O stretching. On the other hand, with adsorbed DEX polymer, -C-O-C stretching at 1095 cm^−1^ appears in a FTIR spectrum in good agreement with the previous study^65^. Characteristic transmission peaks in Ni NPs with PVP were 1460 cm^−1^ for C-H group and 1285–1295 cm^−1^ for C-N bond in PVP derivatives^66^. Compared to pure PVP, slightly shifted C-N peak (from 1285 to 1295 cm^−1^) in Ni NPs with PVP indicates that the formation of coordination bond between Ni and nitrogen atoms from PVP^66^. An IR spectrum of Ni NPs with CMC verified the presence of C-O, C=O, P=O, and P-O stretching at 1410, 1320, 1180 and 1108 cm^−1 ^^67^.

## Discussion

This study successfully examined stabilized Ni NPs by polymers for enhancing the hydration of CO_2_ in the application of geological carbon sequestration. We quantified the influence of ionic strengths on the aggregation of Ni NPs and variations in mass transfer of CO_2_ into the aqueous solution. Although Ni NPs can improve CO_2_ dissolution, their catalytic potential is considerably decreased as the salinity increases due to the aggregation of NPs at the high ionic strength of brine. We found that the performance of Ni NPs could be advanced through the utilization of cationic, nonionic, and anionic polymers. Quantitative comparison of Ni NPs’ catalytic potential in different polymers enables us to determine the best performing polymer for Ni NPs dispersion. The stabilizing ability of nonionic and anionic polymer molecules derived from PVP and CMC at low concentration was found to be superior to the cation polymer for enhancing the Ni NPs’ catalytic activity in brine at high salinity levels. The monovalent cation-enriched in the aqueous solutions could prove conducive to facilitating interactions between anionic polymer molecules from CMC and Ni NPs by providing anchoring sites. These adsorbed polymer layers could reduce the van der Waals attraction force. Collectively, the steric stabilization of Ni NPs by adsorbing anionic polymer has been proved effective in accelerating the hydration of CO_2_, resulting in improving the feasibility and stability of geologic CO_2_ storage in saline aquifers.

## Methods

### Microfluidic devices

The microchannels were fabricated in Poly(dimethylsiloxane) (PDMS; Sylgard 184, Dow Corning Corp., USA) using a standard soft photolithography technique. The geometry designs were printed onto a transparency sheet (25,400 dpi, CAD/ART Services Inc., USA) to fabricate a patterned photomask. After exposure to ultraviolet light through the patterned photomask, patterns were transferred to a layer of negative photoresist (KMPR 1025, Microchem, Newton, MA, USA) coated on a silicon wafer, and a master mold was fabricated. PDMS and its cross-linker were mixed at a ratio of 10:1 (weight per weight) and then degassed using a vacuum pump. The mixture was poured onto the master molds. After curing in an oven at 70 °C for at least 1 hour, the PDMS layers were carefully peeled off from the molds. Two inlet and outlet holes were made using a 1.0 mm diameter biopsy punch (Integra Miltex, Inc., Germany) for connection to a pump (PHD ULTRA 4400, Harvard Apparatus, USA) and a regulator equipped to CO_2_ cylinder (99.9% purity, Airgas, USA) through PTFE (polytetrafluoroethylene) tubing. The PDMS layer and a glass microscope slide (25 × 75 × 1.0 mm, Fisher Scientific, USA) were bonded through oxygen plasma treatment (Harrick Plasma, USA). In a microfluidic device, a 50 μm wide microchannel for gaseous CO_2_ and 100 μm wide microchannels for the liquid and the main channel (where the CO_2_ bubbles and a continuous liquid flow together) were fabricated for generating spherical CO_2_ bubbles. The height of microchannels was 75 μm.

### Experimental configuration

**Figure 6:**
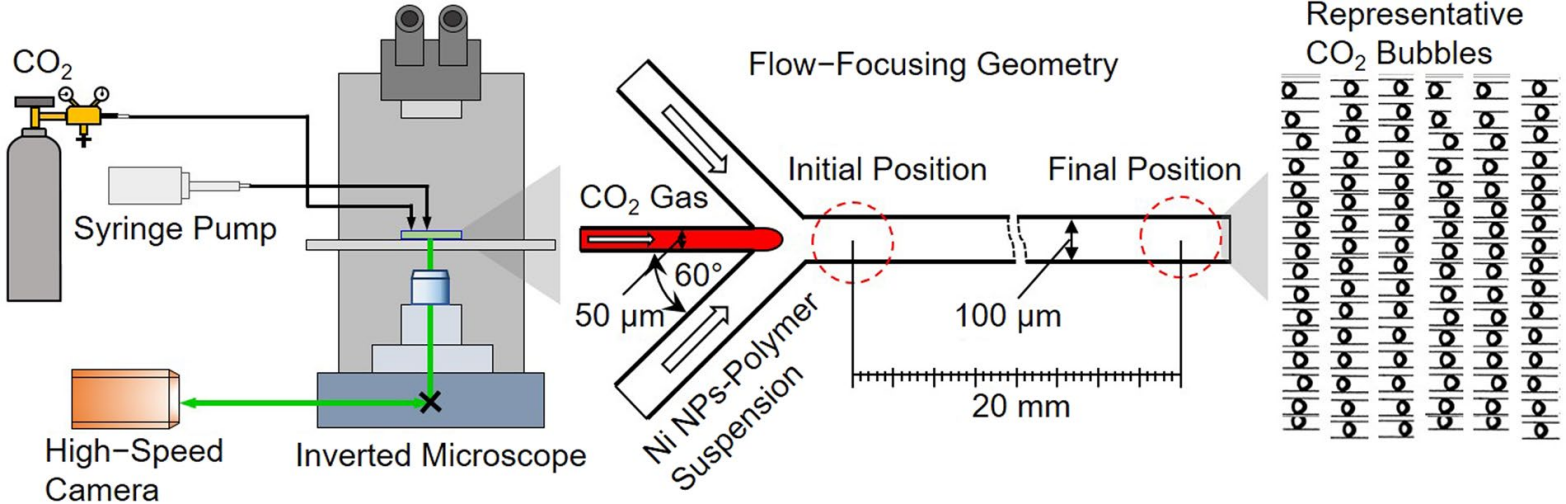
Schematic of test setup (not to scale) and the microfluidic chip of the flow-focusing configuration. An example of collected CO_2_ bubbles is shown for image processing.

Figure 6 shows the overall configuration used for all experiments. The CO_2_ gas and the solutions were injected to a microfluidic chip at a pressure and flow rate of 1 psi and 0.3 mL min^−1^, respectively. A high-speed camera (Fastec IL5S, Fastec Imaging Corp., USA) attached to an inverted microscope (IX73, Olympus Corp., Japan) with a 4x objective lens was used to capture images of CO_2_ bubbles. Since the gas-liquid interface scatters emission light from the microbubbles and appears considerably dark over the surrounding aqueous solution, a binary function in the ImageJ platform was used for estimating the diameter of CO_2_ bubbles. For all experiments, at least fifty images of CO_2_ bubbles were collected at the locations of observation in the microfluidic device. The histograms of CO_2_ bubbles were made to calculate the average initial and final diameters with standard deviations. Then, the percent changes (%∆ = (*D*_*final*_ − *D*_*initial*_)/*D*_*initial*_ × 100) of CO_2_ bubble size caused by CO_2_ dissolution were calculated using the average initial (D_initial_) and final (D_final_) diameters.

### Sample preparation and analysis

Under 100 nm Ni NPs powder (≥99% purity), phosphate buffer powder, and sodium chloride (NaCl, ≥99.5% purity) were purchased from Sigma-Aldrich (USA). One mM phosphate buffer solution (PBS) was prepared using deionized water (18.2 MΩ·cm). Experiments for the effect of the ionic strength on the change in CO_2_ bubble diameter were performed at different salinities in one mM PBS. The mixtures of 30 mg L^−1^ of Ni NPs solutions with different salinities (ionic strengths) of 0%, 2% (0.34), 4% (0.68), 6% (1.03), 8% (1.37), 10% (1.71), 15% (2.57), and 30% (5.13 M) were made to study the salinity effect on the Ni NPs’ catalytic activity. These suspensions were mixed well with a Voltex mixer for 1 min before being injected into the microfluidic device. To make the mixtures of Ni NPs with DEX (Dextran, M.W. 5.0 × 10^5^ g mol^−1^, Alfa Aesar, USA), PVP (Poly(vinyl pyrrolidone), M.W.  5.0 × 10^4^ g mol^−1^, Acros Organics, USA), and CMC (Sodium carboxymethyl cellulose, M.W. 9.0 × 10^4^ g mol^−1^, Acros Organics, USA) at 10% NaCl, Ni NPs suspension, polymeric dispersant solutions, and solution containing salinity were prepared separately at first. Then, they were compounded by the solution to the desired concentrations of 30 mg L^−1^ Ni NPs suspensions with the concentration range of 0.01% to 0.03% of polymer at 10% NaCl. Vortexing these mixtures at 3200 rpm was performed for homogeneity. These mixtures were used to test how efficiently Ni NPs can be dispersed depending on the type and concentration of the polymer. In support of experimental results, the ζ potential of Ni NPs and time-dependent changes in hydrodynamic particle size (d.nm) of Ni NPs with 0.03% of DEX, PVP, and CMC at different salinities (0%, 15%, and 30% NaCl) were measured by a dynamic light scattering system, Zetasizer Nano ZS90 (Malvern Instruments Ltd., UK) using a transparent and disposable zeta cell (DTS 1070, Malvern Instruments Ltd., UK) and a UV-transparent disposable quartz cuvette (Sarstedt AG & Co., Germany), respectively. The mean values of ζ potential of these samples were analyzed from hundred determinations for triplicate samples. A FTIR (Nicolet iS10, Thermo Fisher Scientific, US) was employed to study the interactions between Ni NPs with different polymers. Each drop of 30 mg L^−1^ of Ni NPs suspension with 0.03% of DEX, PVP, and CMC was deposited onto a substrate and dried at room temperature. Then, FTIR spectra of test samples were recorded in the spectral range of 800–4000 cm^−1^.

### Statistical Analysis

All experiments were conducted in triplicate to measure the average initial and final sizes of CO_2_ bubbles. The Pearson’s correlation coefficient was calculated to statistically analyze the dependence of change in CO_2_ bubble diameter at different salinities. The two-sample student’s t-test was used to confirm the statistical significance of the degree of catalytic activity of the Ni NPs-polymer.

## Electronic supplementary material

Supporting Information

The time-dependent changes in CO2 microbubble size in response to the salinity (ionic strength) of the continuous aqueous phase

The time-dependent changes in CO2 microbubble size in response to the salinity (ionic strength) of the continuous aqueous phase in the presence of 30 mg L-1 Ni NPs

The time-dependent changes in CO2 microbubble size in response to stabilized Ni NPs by polymers
