# Supplementary material for: Catalytic activity of nickel nanoparticles stabilized by adsorbing polymers for enhanced carbon sequestration
Source: Sci Rep. 2018 Aug 6;8:11786. doi: 10.1038/s41598-018-29605-1 (PMC6079042; doi:10.1038/s41598-018-29605-1)
Supplement: Supplementary file 1 — Supporting Information [file 41598_2018_29605_MOESM1_ESM.pdf]

Supporting Information

"Catalytic activity of nickel nanoparticles stabilized by adsorbing polymers for enhanced carbon sequestration"

Seokju Seo[1], Gabriela Alvarez Perez[1], Ketan Tewari[2], Xavier Comas[3], and Myeongsub Kim[1,*]

[1]Department of Ocean and Mechanical Engineering, Florida Atlantic University, 777 Glades Road, Boca Raton, FL33431, USA
[2]Department of Mechanical Engineering, Nirma University, Sarkhej-Gandhinagar Highway, Chandlodia, Gota, Ahmedabad, Gujarat 382481, India
[3]Department of Geosciences, Florida Atlantic University, 777 Glades Road, Boca Raton, FL33431, USA
[*]Correspondence and requests for materials should be addressed to M.K. (email: kimm@fau.edu)

This file includes:
1. Legends of Supplementary Movies S1 to S3
2. Supplementary Figures S1 to S3: Histograms of measured diameters of CO_2 bubbles at the initial and final locations

1. Legends of Supplementary Movies S1 to S3

1.1 Supplementary Movie S1:
The time-dependent changes in CO_2 microbubble size in response to the salinity (ionic strength) of the continuous aqueous phase

The video demonstrates that changes in CO_2 bubble size are decreased since the absorption rate of CO_2 gas molecules into the aqueous phase is decreased with an increase in salinity. The CO_2 gas and the prepared solutions were introduced to the microfluidic chip at a pressure and flow rate of 1 psi and 0.3 mL min^{-1}, respectively. The video was recorded at 100 frames per second (fps) but modified to play at 50 fps.

1.2 Supplementary Movie S2:
The time-dependent changes in CO_2 microbubble size in response to the salinity (ionic strength) of the continuous aqueous phase in the presence of 30 mg L^{-1} Ni NPs

This video demonstrates that an increase in salinity results in a significant decrease in catalytic activity of Ni NPs because of their aggregation behavior in high ionic suspension. The CO_2 gas and the prepared solutions were introduced to the microfluidic chip at a pressure and flow rate of 1 psi and 0.3 mL min^{-1}, respectively. The video was recorded at 100 frames per second (fps) but modified to play at 50 fps.

1.3 Supplementary Movie S3:
The time-dependent changes in CO_2 microbubble size in response to stabilized Ni NPs by polymers

This video demonstrates the enhanced catalytic activity of stabilized Ni NPs by polymers at 10% NaCl. The CO_2 gas and the prepared solutions were introduced to the microfluidic chip at a pressure and flow rate of 1 psi and 0.3 mL min^{-1}, respectively. The video was recorded with 100 frames at second (fps) but modified to play at 50 fps.

2. Histograms of measured diameters of CO_2 bubbles at the initial and final locations. The CO_2 bubbles were recorded by high-speed optical microscopy and their sizes were fitted to Gaussian, Lognormal or Lorentzian functions

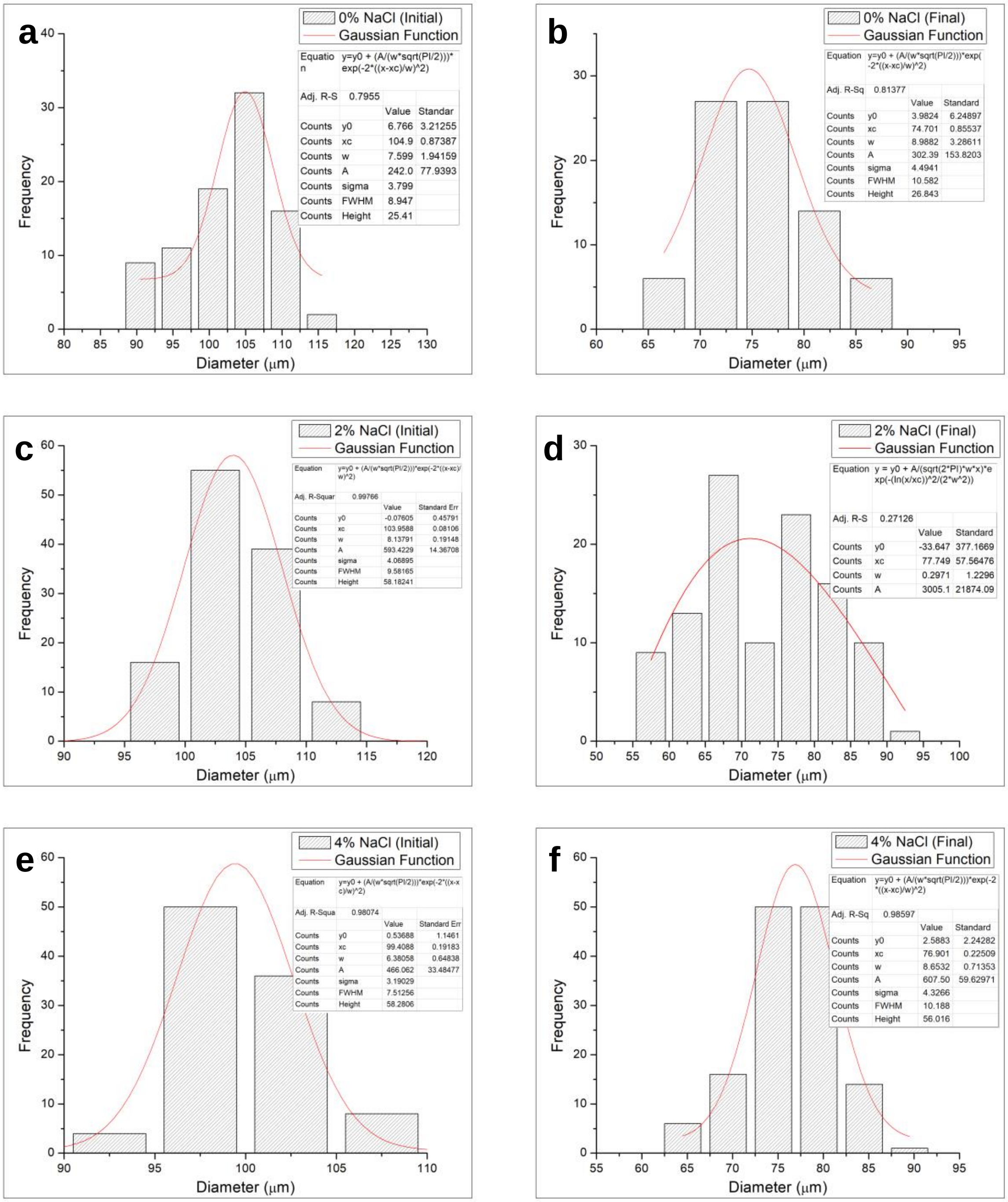

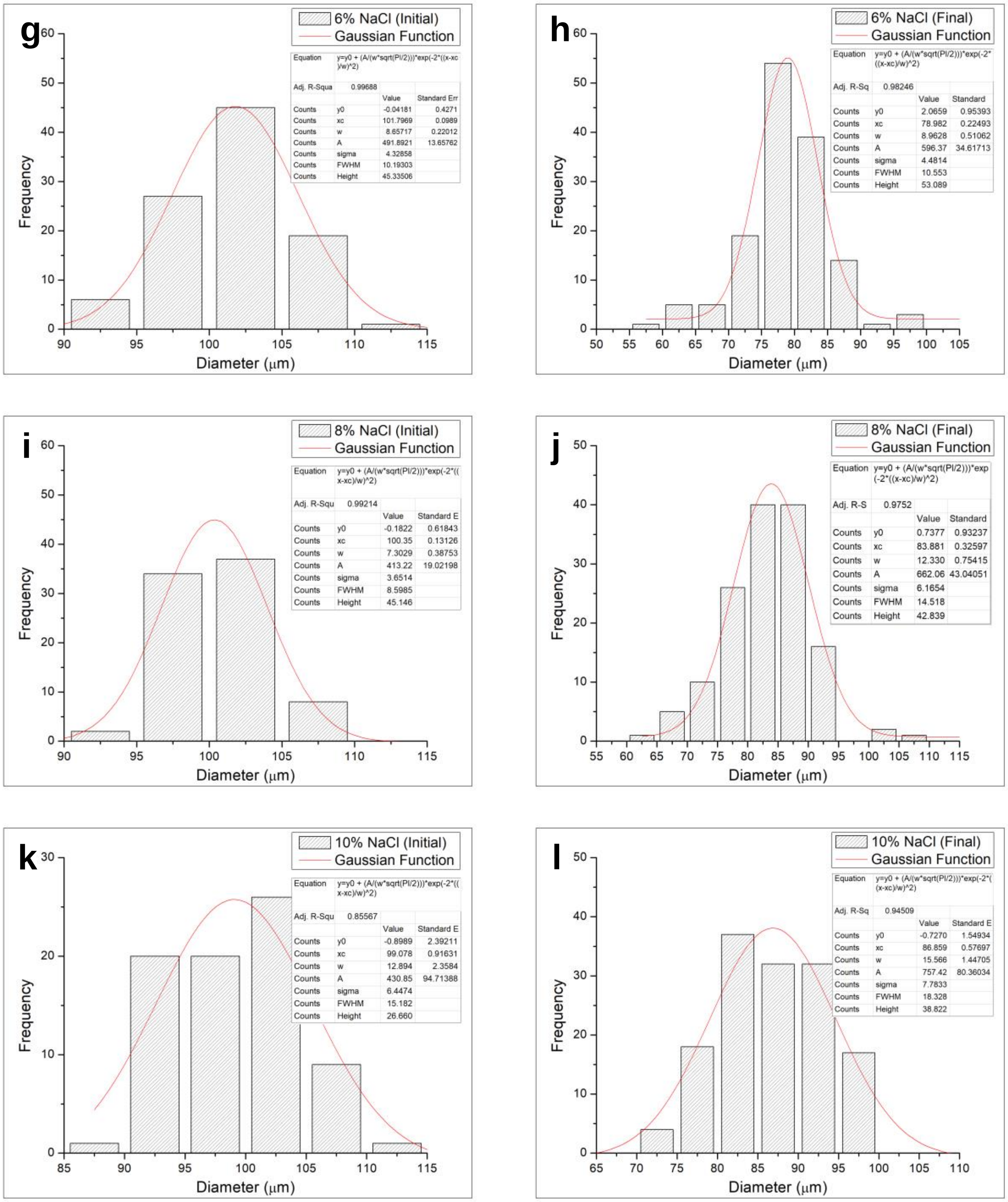
g
6% NaCl (Initial)
Gaussian Function
Equation y=y0 + (A/(w*sqrt(PI/2)))*exp(-2*((x-xc)/w)^2)
Adj. R-Squa 0.99688
Value Standard Err
Counts y0 -0.04181 0.4271
Counts xc 101.7969 0.0989
Counts w 8.65717 0.22012
Counts A 491.8921 13.65762
Counts sigma 4.32858
Counts FWHM 10.19303
Counts Height 45.33506
Frequency
Diameter (μm)
h
6% NaCl (Final)
Gaussian Function
Equation y=y0 + (A/(w*sqrt(PI/2)))*exp(-2*((x-xc)/w)^2)
Adj. R-Sq 0.98246
Value Standard
Counts y0 2.0659 0.95393
Counts xc 78.982 0.22493
Counts w 8.9628 0.51062
Counts A 596.37 34.61713
Counts sigma 4.4814
Counts FWHM 10.553
Counts Height 53.089
Frequency
Diameter (μm)
i
8% NaCl (Initial)
Gaussian Function
Equation y=y0 + (A/(w*sqrt(PI/2)))*exp(-2*((x-xc)/w)^2)
Adj. R-Squ 0.99214
Value Standard E
Counts y0 -0.1822 0.61843
Counts xc 100.35 0.13126
Counts w 7.3029 0.38753
Counts A 413.22 19.02198
Counts sigma 3.6514
Counts FWHM 8.5985
Counts Height 45.146
Frequency
Diameter (μm)
j
8% NaCl (Final)
Gaussian Function
Equation y=y0 + (A/(w*sqrt(PI/2)))*exp(-2*((x-xc)/w)^2)
Adj. R-S 0.9752
Value Standard
Counts y0 0.7377 0.93237
Counts xc 83.881 0.32597
Counts w 12.330 0.75415
Counts A 662.06 43.04051
Counts sigma 6.1654
Counts FWHM 14.518
Counts Height 42.839
Frequency
Diameter (μm)
k
10% NaCl (Initial)
Gaussian Function
Equation y=y0 + (A/(w*sqrt(PI/2)))*exp(-2*((x-xc)/w)^2)
Adj. R-Squ 0.85567
Value Standard E
Counts y0 -0.8989 2.39211
Counts xc 99.078 0.91631
Counts w 12.894 2.3584
Counts A 430.85 94.71388
Counts sigma 6.4474
Counts FWHM 15.182
Counts Height 26.660
Frequency
Diameter (μm)
l
10% NaCl (Final)
Gaussian Function
Equation y=y0 + (A/(w*sqrt(PI/2)))*exp(-2*((x-xc)/w)^2)
Adj. R-Sq 0.94509
Value Standard E
Counts y0 -0.7270 1.54934
Counts xc 86.859 0.57697
Counts w 15.566 1.44705
Counts A 757.42 80.36034
Counts sigma 7.7833
Counts FWHM 18.328
Counts Height 38.822
Frequency
Diameter (μm)

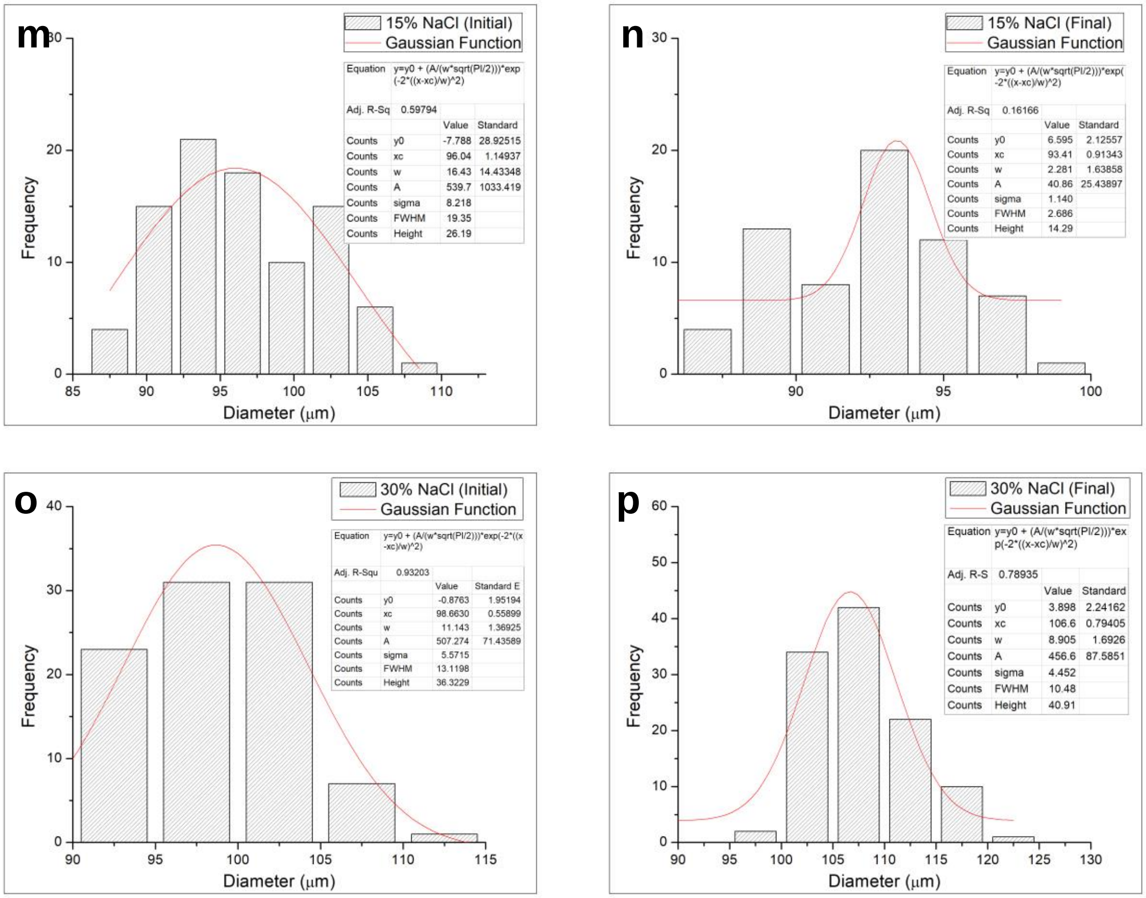

Fig. S1. Histograms of measured diameters of CO_2 bubbles at initial and final positions fitted to a Gaussian function at different salinities (**a** and **b**: 0% NaCl, **c** and **d**: 2% NaCl, **e** and **f**: 4% NaCl, **g** and **h**: 6% NaCl, **i** and **j**: 8% NaCl, **k** and **l**: 10% NaCl, **m** and **n**: 15% NaCl, **o** and **p**: 30% NaCl).

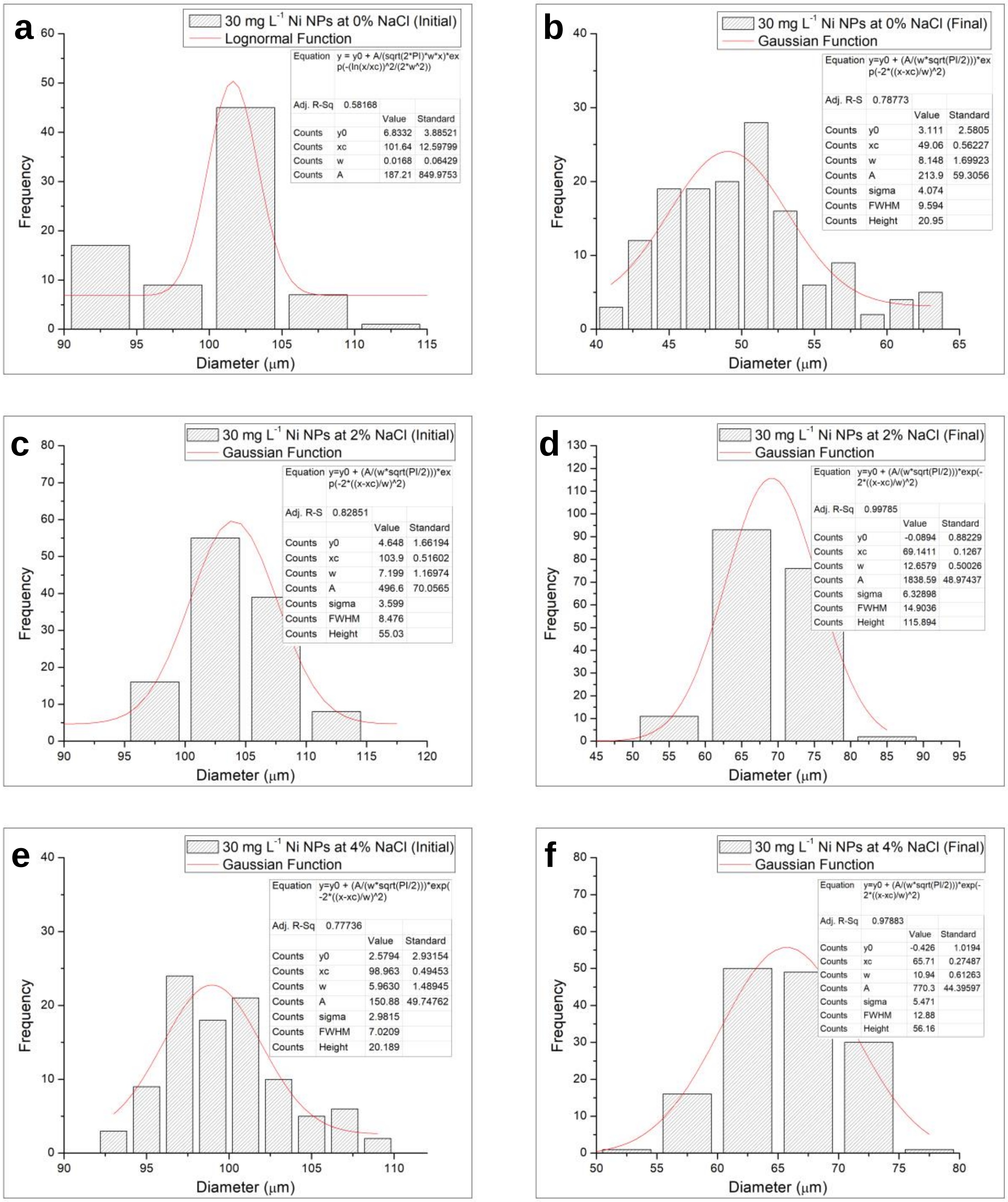
a
30 mg L-1 Ni NPs at 0% NaCl (Initial)
Lognormal Function
Equation y = y0 + A/(sqrt(2*PI)*w*x)*exp(-(ln(x/xc))^2/(2*w^2))
Adj. R-Sq 0.58168
Value Standard
Counts y0 6.8332 3.88521
Counts xc 101.64 12.59799
Counts w 0.0168 0.06429
Counts A 187.21 849.9753
Frequency
Diameter (μm)
b
30 mg L-1 Ni NPs at 0% NaCl (Final)
Gaussian Function
Equation y=y0 + (A/(w*sqrt(PI/2)))*exp(-2*((x-xc)/w)^2)
Adj. R-S 0.78773
Value Standard
Counts y0 3.111 2.5805
Counts xc 49.06 0.56227
Counts w 8.148 1.69923
Counts A 213.9 59.3056
Counts sigma 4.074
Counts FWHM 9.594
Counts Height 20.95
Frequency
Diameter (μm)
c
30 mg L-1 Ni NPs at 2% NaCl (Initial)
Gaussian Function
Equation y=y0 + (A/(w*sqrt(PI/2)))*exp(-2*((x-xc)/w)^2)
Adj. R-S 0.82851
Value Standard
Counts y0 4.648 1.66194
Counts xc 103.9 0.51602
Counts w 7.199 1.16974
Counts A 496.6 70.0565
Counts sigma 3.599
Counts FWHM 8.476
Counts Height 55.03
Frequency
Diameter (μm)
d
30 mg L-1 Ni NPs at 2% NaCl (Final)
Gaussian Function
Equation y=y0 + (A/(w*sqrt(PI/2)))*exp(-2*((x-xc)/w)^2)
Adj. R-Sq 0.99785
Value Standard
Counts y0 -0.0894 0.88229
Counts xc 69.1411 0.1267
Counts w 12.6579 0.50026
Counts A 1838.59 48.97437
Counts sigma 6.32898
Counts FWHM 14.9036
Counts Height 115.894
Frequency
Diameter (μm)
e
30 mg L-1 Ni NPs at 4% NaCl (Initial)
Gaussian Function
Equation y=y0 + (A/(w*sqrt(PI/2)))*exp(-2*((x-xc)/w)^2)
Adj. R-Sq 0.77736
Value Standard
Counts y0 2.5794 2.93154
Counts xc 98.963 0.49453
Counts w 5.9630 1.48945
Counts A 150.88 49.74762
Counts sigma 2.9815
Counts FWHM 7.0209
Counts Height 20.189
Frequency
Diameter (μm)
f
30 mg L-1 Ni NPs at 4% NaCl (Final)
Gaussian Function
Equation y=y0 + (A/(w*sqrt(PI/2)))*exp(-2*((x-xc)/w)^2)
Adj. R-Sq 0.97883
Value Standard
Counts y0 -0.426 1.0194
Counts xc 65.71 0.27487
Counts w 10.94 0.61263
Counts A 770.3 44.39597
Counts sigma 5.471
Counts FWHM 12.88
Counts Height 56.16
Frequency
Diameter (μm)

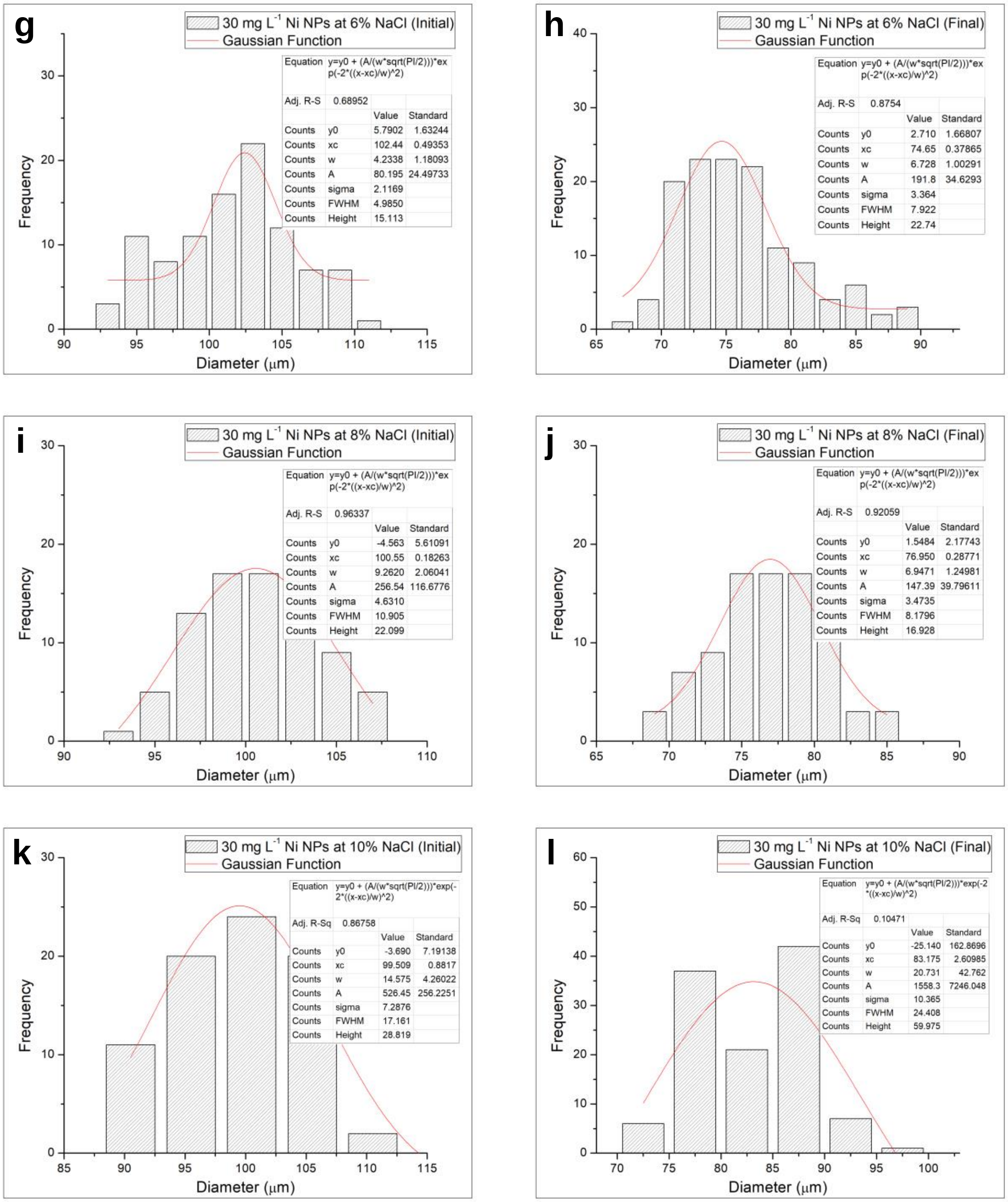
g
30 mg L-1 Ni NPs at 6% NaCl (Initial)
Gaussian Function
Equation y=y0 + (A/(w*sqrt(PI/2)))*exp(-2*((x-xc)/w)^2)
Adj. R-S 0.68952
Value Standard
Counts y0 5.7902 1.63244
Counts xc 102.44 0.49353
Counts w 4.2338 1.18093
Counts A 80.195 24.49733
Counts sigma 2.1169
Counts FWHM 4.9850
Counts Height 15.113
Frequency
Diameter (μm)
h
30 mg L-1 Ni NPs at 6% NaCl (Final)
Gaussian Function
Equation y=y0 + (A/(w*sqrt(PI/2)))*exp(-2*((x-xc)/w)^2)
Adj. R-S 0.8754
Value Standard
Counts y0 2.710 1.66807
Counts xc 74.65 0.37865
Counts w 6.728 1.00291
Counts A 191.8 34.6293
Counts sigma 3.364
Counts FWHM 7.922
Counts Height 22.74
Frequency
Diameter (μm)
i
30 mg L-1 Ni NPs at 8% NaCl (Initial)
Gaussian Function
Equation y=y0 + (A/(w*sqrt(PI/2)))*exp(-2*((x-xc)/w)^2)
Adj. R-S 0.96337
Value Standard
Counts y0 -4.563 5.61091
Counts xc 100.55 0.18263
Counts w 9.2620 2.06041
Counts A 256.54 116.6776
Counts sigma 4.6310
Counts FWHM 10.905
Counts Height 22.099
Frequency
Diameter (μm)
j
30 mg L-1 Ni NPs at 8% NaCl (Final)
Gaussian Function
Equation y=y0 + (A/(w*sqrt(PI/2)))*exp(-2*((x-xc)/w)^2)
Adj. R-S 0.92059
Value Standard
Counts y0 1.5484 2.17743
Counts xc 76.950 0.28771
Counts w 6.9471 1.24981
Counts A 147.39 39.79611
Counts sigma 3.4735
Counts FWHM 8.1796
Counts Height 16.928
Frequency
Diameter (μm)
k
30 mg L-1 Ni NPs at 10% NaCl (Initial)
Gaussian Function
Equation y=y0 + (A/(w*sqrt(PI/2)))*exp(-2*((x-xc)/w)^2)
Adj. R-Sq 0.86758
Value Standard
Counts y0 -3.690 7.19138
Counts xc 99.509 0.8817
Counts w 14.575 4.26022
Counts A 526.45 256.2251
Counts sigma 7.2876
Counts FWHM 17.161
Counts Height 28.819
Frequency
Diameter (μm)
l
30 mg L-1 Ni NPs at 10% NaCl (Final)
Gaussian Function
Equation y=y0 + (A/(w*sqrt(PI/2)))*exp(-2*((x-xc)/w)^2)
Adj. R-Sq 0.10471
Value Standard
Counts y0 -25.140 162.8696
Counts xc 83.175 2.60985
Counts w 20.731 42.762
Counts A 1558.3 7246.048
Counts sigma 10.365
Counts FWHM 24.408
Counts Height 59.975
Frequency
Diameter (μm)

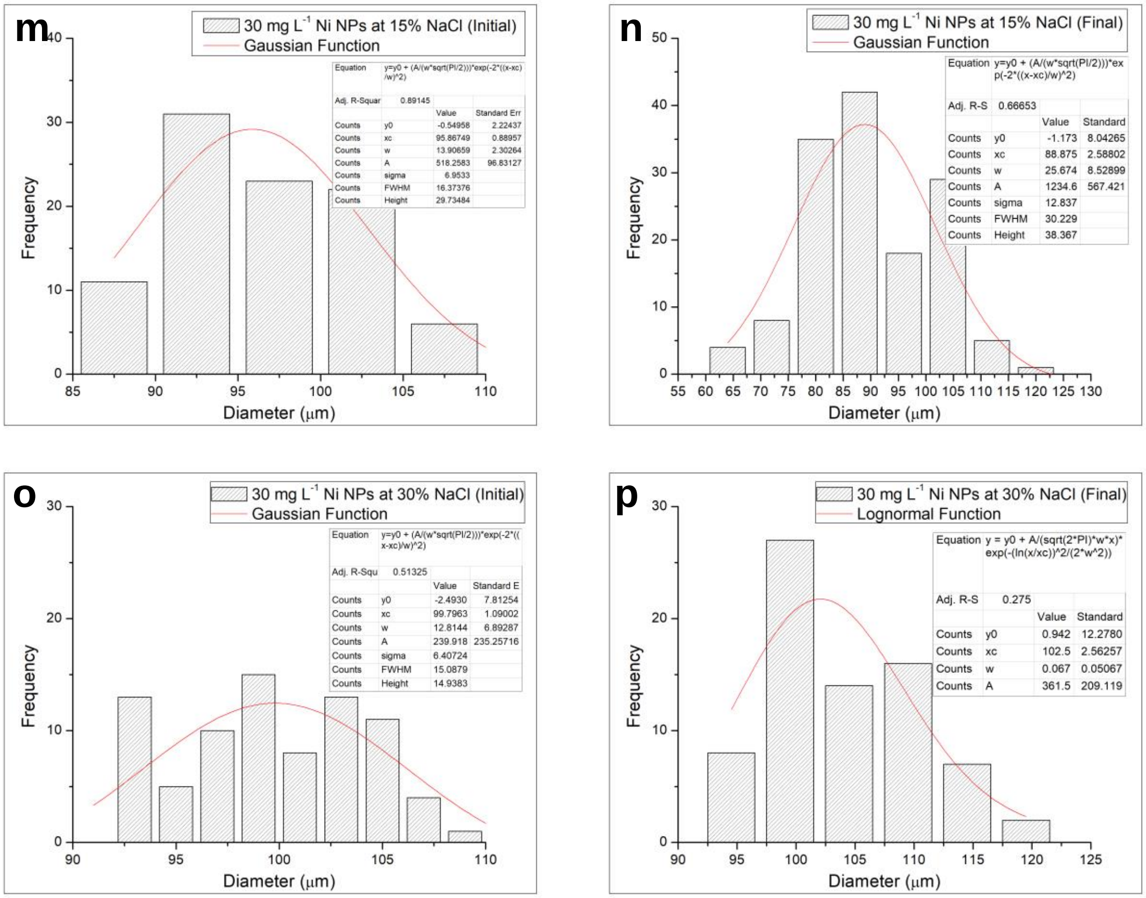

Fig. S2. Histograms of measured diameters of CO_2 bubbles at initial and final positions fitted to Gaussian or Lognormal functions in the presence of 30 mg L^{-1} Ni NPs at different salinities (**a** and **b**: 0% NaCl, **c** and **d**: 2% NaCl, **e** and **f**: 4% NaCl, **g** and **h**: 6% NaCl, **i** and **j**: 8% NaCl, **k** and **l**: 10% NaCl, **m** and **n**: 15% NaCl, **o** and **p**: 30% NaCl).

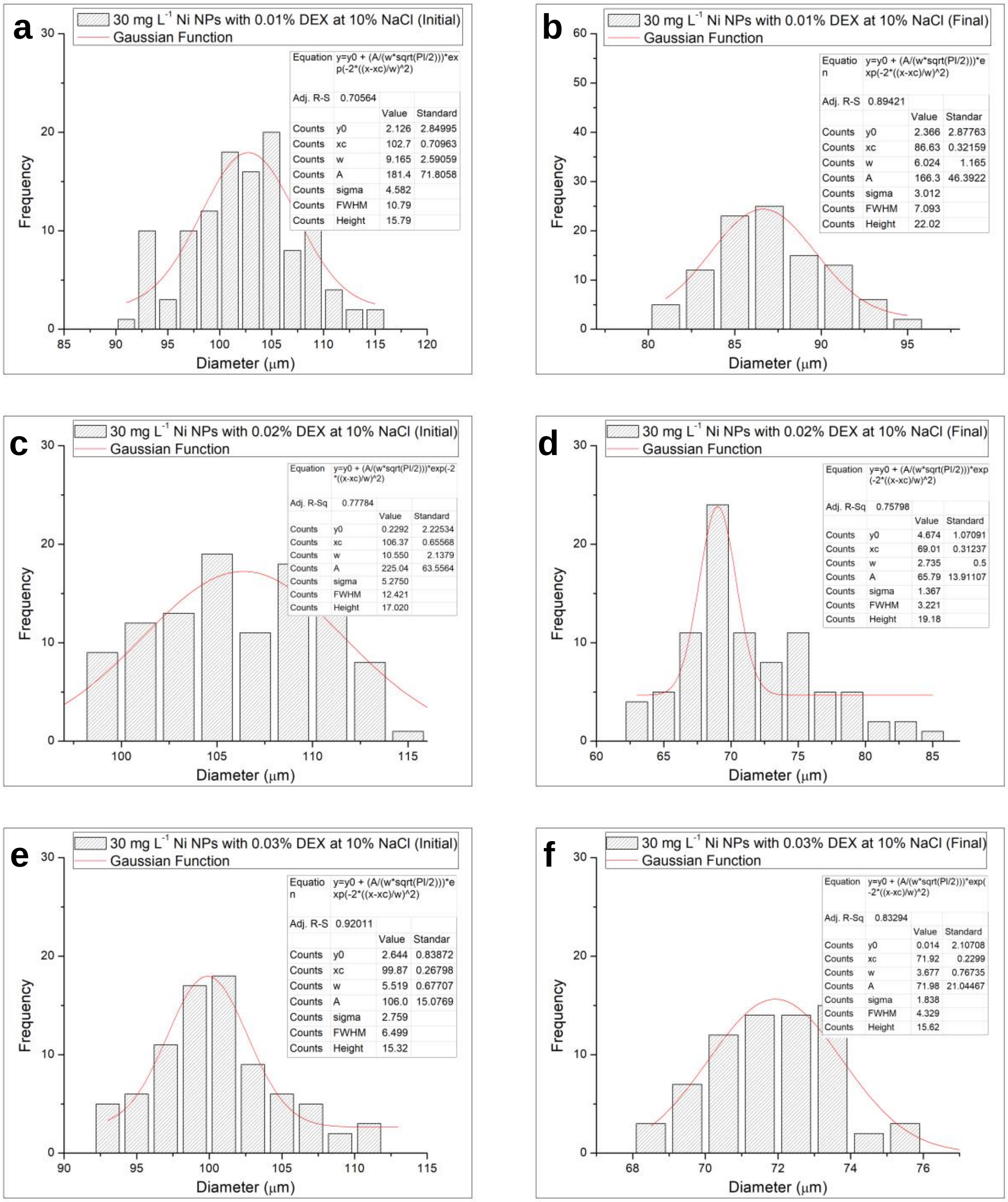
a
30 mg L-1 Ni NPs with 0.01% DEX at 10% NaCl (Initial)
Gaussian Function
Equation y=y0 + (A/(w*sqrt(PI/2)))*exp(-2*((x-xc)/w)^2)
Adj. R-S 0.70564
Value Standard
Counts y0 2.126 2.84995
Counts xc 102.7 0.70963
Counts w 9.165 2.59059
Counts A 181.4 71.8058
Counts sigma 4.582
Counts FWHM 10.79
Counts Height 15.79
Frequency
Diameter (μm)
b
30 mg L-1 Ni NPs with 0.01% DEX at 10% NaCl (Final)
Gaussian Function
Equation y=y0 + (A/(w*sqrt(PI/2)))*exp(-2*((x-xc)/w)^2)
Adj. R-S 0.89421
Value Standar
Counts y0 2.366 2.87763
Counts xc 86.63 0.32159
Counts w 6.024 1.165
Counts A 166.3 46.3922
Counts sigma 3.012
Counts FWHM 7.093
Counts Height 22.02
Frequency
Diameter (μm)
c
30 mg L-1 Ni NPs with 0.02% DEX at 10% NaCl (Initial)
Gaussian Function
Equation y=y0 + (A/(w*sqrt(PI/2)))*exp(-2*((x-xc)/w)^2)
Adj. R-Sq 0.77784
Value Standard
Counts y0 0.2292 2.22534
Counts xc 106.37 0.65568
Counts w 10.550 2.1379
Counts A 225.04 63.5564
Counts sigma 5.2750
Counts FWHM 12.421
Counts Height 17.020
Frequency
Diameter (μm)
d
30 mg L-1 Ni NPs with 0.02% DEX at 10% NaCl (Final)
Gaussian Function
Equation y=y0 + (A/(w*sqrt(PI/2)))*exp(-2*((x-xc)/w)^2)
Adj. R-Sq 0.75798
Value Standard
Counts y0 4.674 1.07091
Counts xc 69.01 0.31237
Counts w 2.735 0.5
Counts A 65.79 13.91107
Counts sigma 1.367
Counts FWHM 3.221
Counts Height 19.18
Frequency
Diameter (μm)
e
30 mg L-1 Ni NPs with 0.03% DEX at 10% NaCl (Initial)
Gaussian Function
Equation y=y0 + (A/(w*sqrt(PI/2)))*exp(-2*((x-xc)/w)^2)
Adj. R-S 0.92011
Value Standar
Counts y0 2.644 0.83872
Counts xc 99.87 0.26798
Counts w 5.519 0.67707
Counts A 106.0 15.0769
Counts sigma 2.759
Counts FWHM 6.499
Counts Height 15.32
Frequency
Diameter (μm)
f
30 mg L-1 Ni NPs with 0.03% DEX at 10% NaCl (Final)
Gaussian Function
Equation y=y0 + (A/(w*sqrt(PI/2)))*exp(-2*((x-xc)/w)^2)
Adj. R-Sq 0.83294
Value Standard
Counts y0 0.014 2.10708
Counts xc 71.92 0.2299
Counts w 3.677 0.76735
Counts A 71.98 21.04467
Counts sigma 1.838
Counts FWHM 4.329
Counts Height 15.62
Frequency
Diameter (μm)

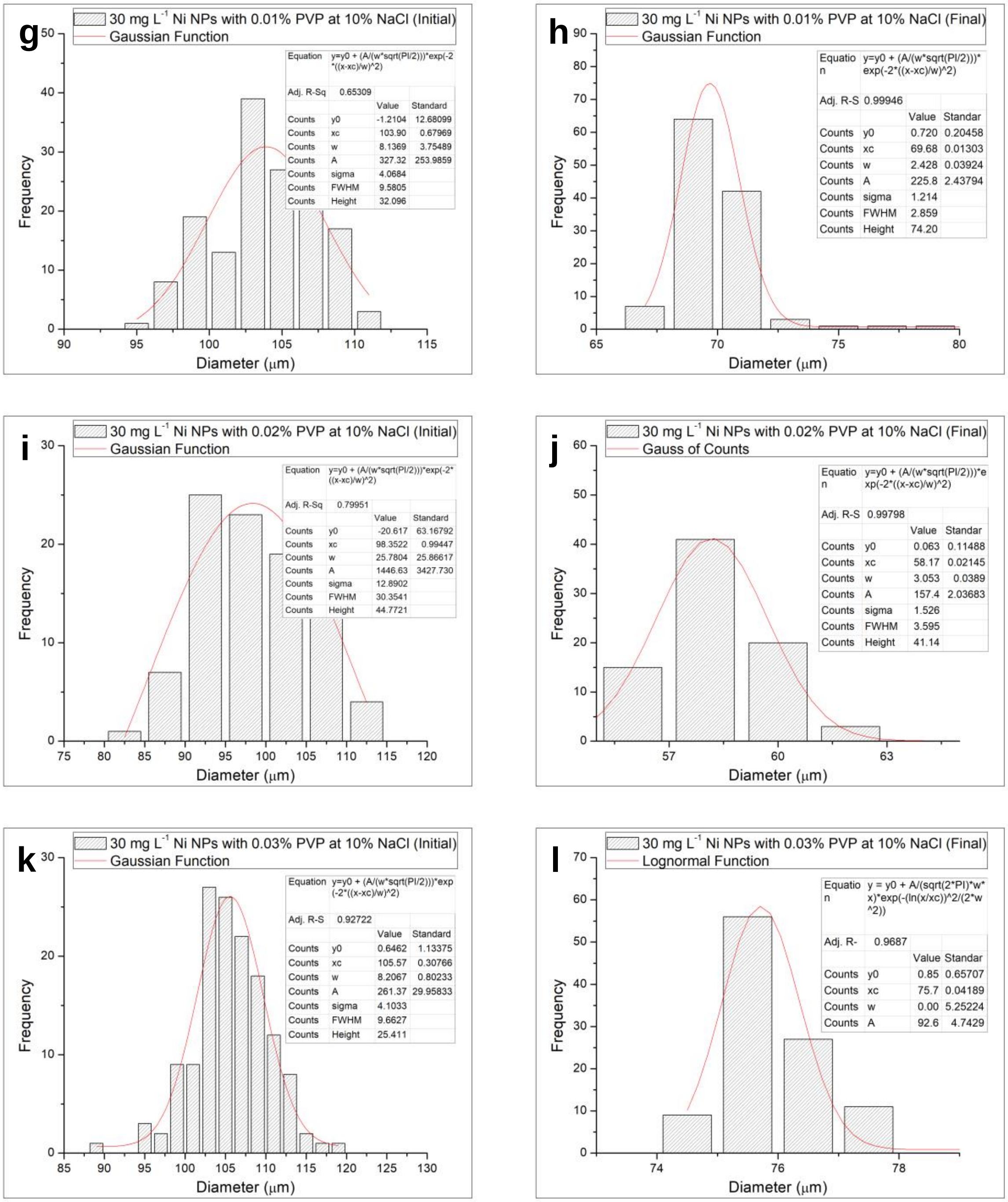
g
30 mg L-1 Ni NPs with 0.01% PVP at 10% NaCl (Initial)
Gaussian Function
Equation y=y0 + (A/(w*sqrt(PI/2)))*exp(-2*((x-xc)/w)^2)
Adj. R-Sq 0.65309
Value Standard
Counts y0 -1.2104 12.68099
Counts xc 103.90 0.67969
Counts w 8.1369 3.75489
Counts A 327.32 253.9859
Counts sigma 4.0684
Counts FWHM 9.5805
Counts Height 32.096
Frequency
Diameter (μm)
h
30 mg L-1 Ni NPs with 0.01% PVP at 10% NaCl (Final)
Gaussian Function
Equation y=y0 + (A/(w*sqrt(PI/2)))*exp(-2*((x-xc)/w)^2)
Adj. R-S 0.99946
Value Standar
Counts y0 0.720 0.20458
Counts xc 69.68 0.01303
Counts w 2.428 0.03924
Counts A 225.8 2.43794
Counts sigma 1.214
Counts FWHM 2.859
Counts Height 74.20
Frequency
Diameter (μm)
i
30 mg L-1 Ni NPs with 0.02% PVP at 10% NaCl (Initial)
Gaussian Function
Equation y=y0 + (A/(w*sqrt(PI/2)))*exp(-2*((x-xc)/w)^2)
Adj. R-Sq 0.79951
Value Standard
Counts y0 -20.617 63.16792
Counts xc 98.3522 0.99447
Counts w 25.7804 25.86617
Counts A 1446.63 3427.730
Counts sigma 12.8902
Counts FWHM 30.3541
Counts Height 44.7721
Frequency
Diameter (μm)
j
30 mg L-1 Ni NPs with 0.02% PVP at 10% NaCl (Final)
Gauss of Counts
Equation y=y0 + (A/(w*sqrt(PI/2)))*exp(-2*((x-xc)/w)^2)
Adj. R-S 0.99798
Value Standar
Counts y0 0.063 0.11488
Counts xc 58.17 0.02145
Counts w 3.053 0.0389
Counts A 157.4 2.03683
Counts sigma 1.526
Counts FWHM 3.595
Counts Height 41.14
Frequency
Diameter (μm)
k
30 mg L-1 Ni NPs with 0.03% PVP at 10% NaCl (Initial)
Gaussian Function
Equation y=y0 + (A/(w*sqrt(PI/2)))*exp(-2*((x-xc)/w)^2)
Adj. R-S 0.92722
Value Standard
Counts y0 0.6462 1.13375
Counts xc 105.57 0.30766
Counts w 8.2067 0.80233
Counts A 261.37 29.95833
Counts sigma 4.1033
Counts FWHM 9.6627
Counts Height 25.411
Frequency
Diameter (μm)
l
30 mg L-1 Ni NPs with 0.03% PVP at 10% NaCl (Final)
Lognormal Function
Equation y = y0 + A/(sqrt(2*PI)*w*x)*exp(-(ln(x/xc))^2/(2*w^2))
Adj. R- 0.9687
Value Standar
Counts y0 0.85 0.65707
Counts xc 75.7 0.04189
Counts w 0.00 5.25224
Counts A 92.6 4.7429
Frequency
Diameter (μm)

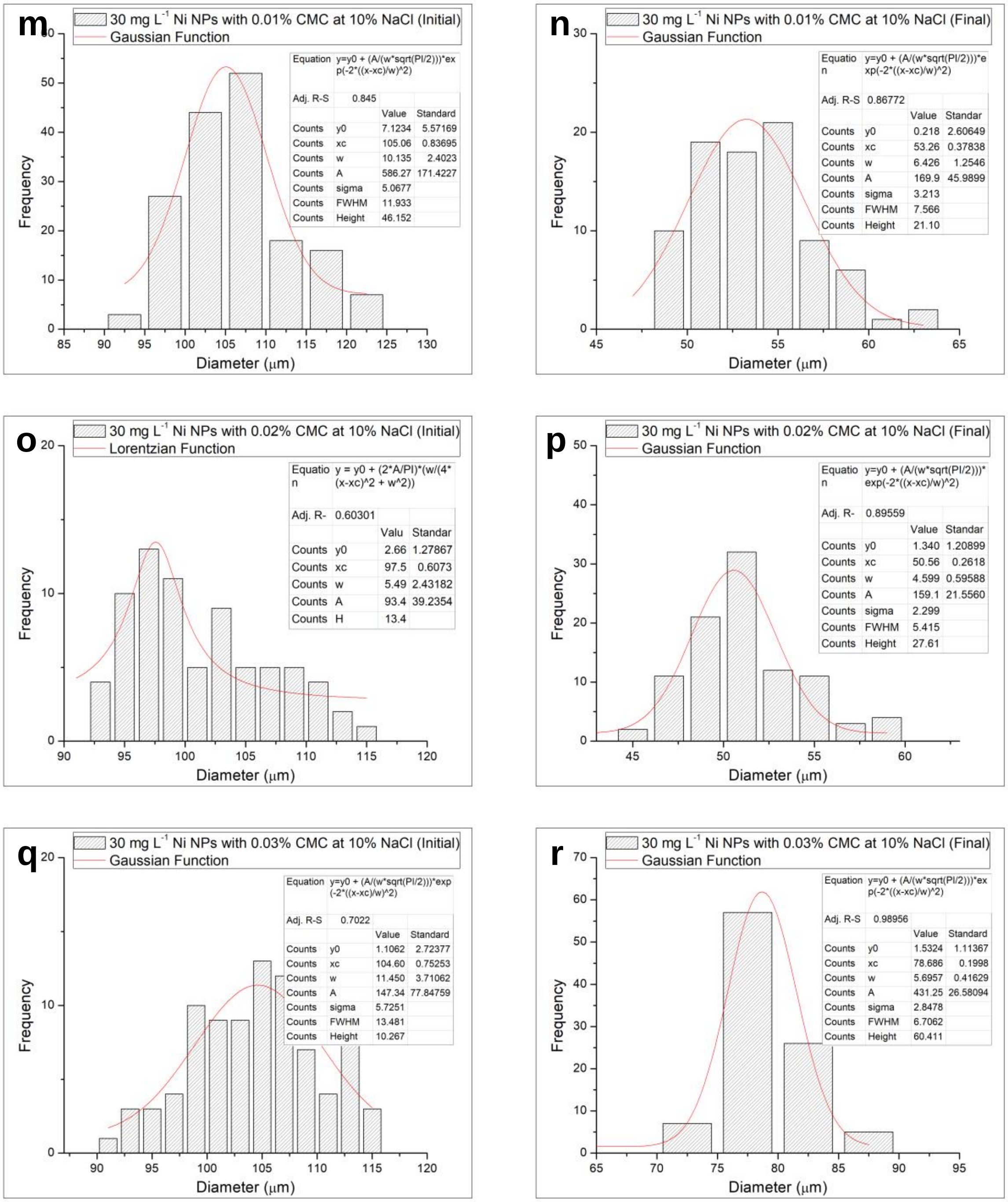

Fig. S3. Histograms of measured diameters of CO_2 bubbles at initial and final positions fitted to Gaussian, Lorentzian or Lognormal functions in the presence of Ni NPs stabilized by 0.01%, 0.02%, and 0.03% of DEX, PVP, and CMC at 10% NaCl. (**a** and **b**: 0.01% DEX, **c** and **d**: 0.02% DEX, **e** and **f**: 0.03% DEX, **g** and **h**: 0.01% PVP, **i** and **j**: 0.02% PVP, **k** and **l**: 0.03% PVP, **m** and **n**: 0.01% CMC, **o** and **p**: 0.02% CMC, **q** and **r**: 0.03% CMC).
